# Correction: Using moss as a bio-indicator to evaluate soil quality in litchi orchard

**DOI:** 10.1371/journal.pone.0319554

**Published:** 2025-02-13

**Authors:** The PLOS One Staff

## Notice of republication

This article was republished on Januray 16, 2025, to correct an error in the author list: Shangdong Yang was erroneously displayed as Shangdong Yang Yang. The publisher apologises for the error. Please download this article again to view the correct version. The originally published, uncorrected article and the republished, corrected article are provided here for reference.

## Supporting information

S1 File Originally published, uncorrected article. (PDF)

S2 File Republished, corrected article. (PDF)
