# Supplementary material for: Correction: Using moss as a bio-indicator to evaluate soil quality in litchi orchard
Source: PLoS One. 2025 Feb 13;20(2):e0319554. doi: 10.1371/journal.pone.0319554 (PMC11825037; doi:10.1371/journal.pone.0319554)
Supplement: S2 File — (PDF) [file pone.0319554.s002.pdf]

RESEARCH ARTICLE

Using moss as a bio-indicator to evaluate soil quality in litchi orchard

Siyu Chen[1], Yan Sun[1], Da Yang[1], Shandong Yang[1]*, Tian Liang[2], Hongwei Tan[2]*

1 Guangxi Key Laboratory of Agro-environment and Agro-products Safety, National Demonstration Center for Experimental Plant Science Education Guangxi Agricultural College, Guangxi University, Nanning, Guangxi, P.R. China, **2** Guangxi Key Laboratory of Sugarcane Genetic Improvement, Guangxi Academy of Agricultural Sciences, Nanning, Guangxi, P.R. China

* 924433816@qq.com (SY); hongwei_tan@163.com (HT)

Abstract

Moss, was frequently found growing in litchi orchards. However, less known about whether it can be used as a visual bio-indicator for evaluating soil fertility and health. Therefore, soil chemical and biological properties, microbial community structures and the metabolic functions of microbes in soils between moss- and non-moss-growth areas were analyzed using traditional and high-throughput sequencing technologies. The results showed that pH and the contents of and available phosphorus (AP) in moss growth areas were significantly lower than those of non-moss growth areas, but the contents of alkali-hydrolyzable nitrogen (AN) and available potassium (AK) were significantly increased. In comparison with the soil of the non-moss-growth area, the abundances of hypotrophic microorganisms, such as Chloroflexi, Cyanobacteria and WPS-2 enriched in the soil of the moss-grown area. Moreover, the proportions of eutrophic microorganisms, such as Proteobacteria and Firmicutes also declined in the soils of the moss-growth area. Furthermore, the metabolic pathways of soil bacteria and fungi were also degraded in the moss-growth area. All above results indicated that not only lower soil fertility, but also soil microbial diversity also declined in moss growth area which compared to those of non-moss growth area. In one word, moss can be considered using as a visual bio-indicator for representing soil degradation in litchi orchards.

OPEN ACCESS

Citation: Chen S, Sun Y, Yang D, Yang S, Liang T, Tan H (2022) Using moss as a bio-indicator to evaluate soil quality in litchi orchard. PLoS ONE 17(12): e0278303. https://doi.org/10.1371/journal.pone.0278303

Editor: Upendra Kumar, ICAR-National Rice Research Institute, INDIA

Received: August 3, 2022

Accepted: November 14, 2022

Published: December 30, 2022

Copyright: © 2022 Chen et al. This is an open access article distributed under the terms of the Creative Commons Attribution License, which permits unrestricted use, distribution, and reproduction in any medium, provided the original author and source are credited.

Data Availability Statement: Raw data for bacterial and fungal sequence were depositied in the NCBI Sequence Read Archive (SRA) database under accession number PRJNA863626 and PRJNA863722 respectively.

Funding: This research was funded by the National Key R & D Program of China (2020YFD1000600) and National Natural Science Foundation of China (No. 31860547). The funders had no role in study design, data collection and analysis, decision to publish, or preparation of the manuscript.

Introduction

Litchi (*Litchi chinensis* Sonn.) is a tropical and subtropical fruit, and it is mainly distributed in latitudes between 23˚ and 27˚, such as in southern China, northern Vietnam, and Malaysia [1]. At present, it is widely grown in more than 20 countries around the world, and it has become has become a popular fruit [2]. However, its yield fluctuates year after year, and it is difficult to achieve yield stability [3]. In severe cases, even zero production can be found [4]. Insufficient nutrition is one of the important reasons for low litchi yields [5]. Previous studies have found that the contents of nitrogen (N) and potassium(K) in soil always induce low litchi yields [6]. In addition, litchi development requires abundant phosphorus (P), K, and calcium (Ca) during late fruit development [7]. As is well known, K plays an important role in fruit development, quality formation, and stress and disease resistances [8–10]. The amount of

Competing interests: The authors have declared that no competing interests exist.

available P in soil is positively correlated with the amount of P in litchi leaves, which affects the litchi yield. An inappropriate ratio of N to K results in fewer flower spikes and severe flower and fruit drop; soil fertility is an important factor in regulating litchi yields [11, 12].

However, soil fertility cannot be immediately assessed by producers in practice. In particular, it is difficult to evaluate soil quality during the production of fruits, including litchi, citrus, and most other fruits. Recently, we found that moss could grow on the soil surface like a carpet in a litchi orchard (Fig 1A and 1B).

Previous studies have confirmed that moss is an important ground covering for degraded or damaged ecosystems, and it plays a vital role in their ecological properties [13]. Moss affects soil properties; in particular, it alters soil fertility through the accumulation of total N, K or alkali-soluble N, available K, P, and organic matter in the soil [14, 15]. For example, moss enriches the P in soil during moss growth [16]; i.e., the more P is absorbed by mosses, the less P is left in the soil [17]. Meanwhile, moss is very sensitive to its environment and easily alters the water content and the nutrients in the environment with its growth [18–21]. Previous studies have considered moss as a bio-indicator for soil and water conservation, vegetation changes, environmental monitoring, and forest integrity [22, 23].

In addition, soil microorganisms are important parts of ecosystems; not only they are closely linked to the energy cycle of terrestrial ecosystems, but they also participate in the cycling of material above and below the ecosystem floor, leading to the stability of the terrestrial ecosystem [24]. Bacteria are the most abundant taxa in soil, accounting for 70–90% of all soil microorganisms [25], and they play an important role in nutrient cycling and in maintaining soil health [26]. Fungi are also key members of soil microbiota; they are a major source of soil microbial biomass and are important drivers in maintaining cycles of matter in ecology [27]. The soil microbial community structure reflects the evolution of the ecosystem function, which relates to soil properties and environmental factors [9, 28]. It has been confirmed that changes in soil properties often lead to alterations in the microbial community structure [29, 30]. Moreover, soil microorganisms are very sensitive to the changes in their living environment, including soil nutrients [31], pH [32], soil physical properties [33], and plant diversity, among which soil nutrients and water content are the two most important factors affecting the diversity and function of soil microorganisms [34]. Additionally, soil microbial biomass C, N, and P are important indicators of soil health [35], and they can be used to evaluate soil quality,

(a) (b)

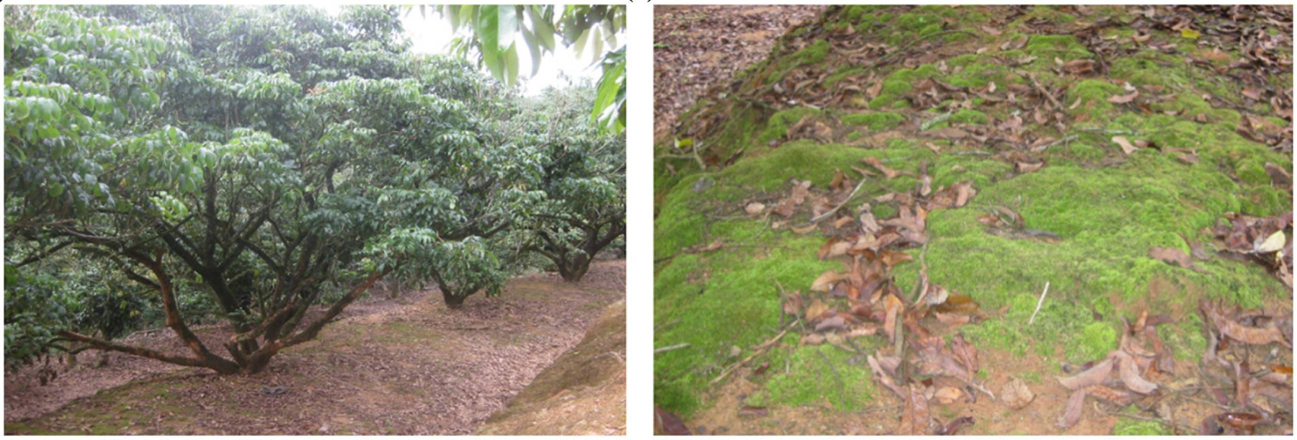

Fig 1. Moss carpets forming on the soil surface in litchi orchard.

https://doi.org/10.1371/journal.pone.0278303.g001

and crop productivity [36]. Soil enzyme activity is also an important index for soil quality assessment [37], and it can reflect the energy metabolism and material cycle in soil. As is well-known, the soil microbial community structure is sensitive to environmental changes, and also can be used as a bio-indicator to reflect soil nutrient status and health. However, the bio-indicators of soil enzymes and soil microbial community structure, etc., are need to be analyzed firstly in lab, they are all not the visual indicators for soil quality.

Till now, studies on the relationship between moss and microbial community have focused on bacteria [38], Endophytic bacteria [39]. But minimal information is available on moss whether can be used as a visual bio- indicator for reflecting soil quality. Therefore, our aim here is to elucidate how differences of the soil biological properties, including soil enzymes, soil microbial biomass and the soil microbial compositions and their metabolic functions between moss and non-moss growth areas in a litchi orchard.

Materials and methods

Field site description

The experimental site is located in a litchi orchard in Gao Li Village, Qinzhou City, Guangxi Zhuang Autonomous Region (109˚16′31″ E, 22˚12′30″ N), China. This area has a hilly topography and a subtropical monsoon climate; its average annual precipitation, temperature and frost-free period are 1658 mm, 22˚C and 348 d, respectively. Min and Max temperatures are 0˚C and 32˚C, respectively.

Soil sampling

The soil samples were collected in a Litchi orchard at Gaoli village (E: 109˚1621, N: 22˚1230), Qinzhou city of Guangxi Province, China on 26 May 2018. Feizixiao, a widely planted litchi cultivar, with 12-years old trees in the orchards under conventional managements were used in this study. And each litchi tree was applied 0.6 kg nitrogen, 0.18 kg phosphorus and 0.6 kg potassium using urea (N: 46.0%), superphosphate (P_2O_5:12.0%) and potassium chloride (K_2O: 60.0%), respectively.

Impurities on the soil surface were lightly removed, and soil samples from moss (hereafter abbreviated as M) and non-moss (hereafter abbreviated as L) areas were both carefully collected using a sterilized auger at a depth of 20 cm. Then, the soil samples were put into sterilized plastic bags and placed in an ice box with ice bags. After being transferred to the laboratory, soil samples were sieved through a 2 mm mesh stainless steel sieve, and then they were stored in a refrigerator at 4˚C for immediate analysis or were stored at −80˚C for later use. Meanwhile, parts of the soil samples were air-dried at room temperature for soil chemical analyses.

Soil chemical and biological properties

First, soil pH was measured using a pH meter (soil–water ratio 1:2.5). Total nitrogen (TN) was determined using the semimicro-Kjeldahl method [40]. Total phosphorus (TP) was determined by the alkali fusion-molybdenum anti-colorimetric method [41]. Total potassium (TK) was determined by alkali fusion-flame spectrophotometry [41]. Available P was determined using acid-fluoride solutions method [42], and available N, K were determined alkali diffusion method and flame photometry respectively [41].

Soil microbial biomass

The soil microbial biomass carbon (MBC) were determined using the 0.5 M K_2S0_4 chloroform fumigation–extraction method as described by Vance et al. [43]. The soil microbial biomass

nitrogen (MBN) was measured using the Ninhydrin-reactive nitrogen measurements of microbial biomass in 0 5 M K_2SO_4 soil extracts method described by Joergensen et al. [44]. The soil microbial biomass phosphorus (MBP) was determined using fumigation-extraction with 0.5 M NaHCO3 at pH 8.5 as described by Powlson et al [36].

Soil enzyme activities soil microbial biomass

β-Glucosidase activity was determined using colorimetric estimation of ρ-nitrophenol released by β-glucosidase activity when soil is incubated in McIlvaine buffer (pH 4.8) with ρnitrophenyl β-glucoside and toluene at 30˚C for 1 hr method as described by Hayano et al. [45]. Amino-peptidase activity was measured using the using benzyloxycarbonyl phenylalanyl leucine as substrate, separated from the proteases by precipitation with 0.1 m $CaCl_2$. And using Tris-borate buffer extracts of a highly-organic method as described by Ladd et al. [46]. Acid phosphatase activity was determined using the universal buffer of pH 6.5, which containing 50 mM p-nitrophenyl phosphateat 37˚C for 1 h. The potential enzyme activities were defined as μg of p-nitrophenol (pNP) released by per g of soil within 1 h chloroform fumigation–extraction method as described by Tabatabai et al. [47].

Analysis of soil microbial community structure

Microbial community genomic DNA was extracted from samples using an E.Z.N.A.® soil DNA Kit (Omega Bio-tek, Norcross, GA, USA) according to the manufacturer's instructions. The DNA extract was checked on 1% agarose gel, and the DNA concentrations and purity were determined using a NanoDrop 2000 UV–vis spectrophotometer (Thermo Scientific, Wilmington, NC, USA). PCR amplification and sequencing of the total DNA extracted from the rhizosphere soil samples were performed by Shanghai Majorbio Bio-pharm Technology Co., Ltd. (Shanghai, China), while PCR amplification was performed using an ABI GeneAmp® type 9700 (ABI, CA, USA) and the products were recovered by 2% agarose gel electrophoresis, purified by an Axy PrepDNA gel recovery kit (AXYGEN) (Axygen Biosciences, Union City, CA, USA), eluted by Tris HCl, and quantified by the QuantiFluor™-ST (Promega, USA). Blue Fluorescence quantitative system (Promega). The purified amplicons were pooled in equimolar quantities and were paired-end sequenced (2 × 300) on the Illumina MiSeq platform (Illumina, San Diego, CA, USA) according to the standard protocols of Majorbio Bio-Pharm Technology Co. Ltd. (Shanghai, China). Raw reads were deposited in the NCBI Sequence Read Archive (SRA) database (Table 1).

PICRUSt gene function prediction

PICRUSt was used to remove the effect of the number of copies of the 16S marker gene in the genome of the species and to standardize the OTU (Operational Taxonomic Unit) abundance table, using the green gene ID corresponding to each OTU. The KEGG Orthology (KO) information and COG family information corresponding to each OTU were obtained; then, the abundance of each COG and KO could be calculated. The COG database was parsed against

Table 1. Sequencing type and primer sequence.

Source	Primer Name	Primer Sequence	Sequencing Platform
Soil bacteria	338F	5′-ACTCCTACGGGAGGCAGCAG-3′	PE300
	806R	5′-GGACTACHVGGGTWTCTAAT-3′	
ITS	ITS1F	5′-CTTGGTCATTTAGAGGAAGTAA-3′	PE250
	ITS2F	5′-GCTGCGTTCTTCATCGATGC-3′	

https://doi.org/10.1371/journal.pone.0278303.t001

Table 2. Soil physicochemical properties between moss- (M) and non-moss growth (L) areas in litchi orchard.

Samples	SWC	pH	TN (%)	TP (g/kg)	TK (%)	AN (mg/kg)	AP (mg/kg)	AK (mg/kg)
L	2.13 ± 0.31 a	4.8 ± 0.01 a	0.12 ± 0.01 a	80.27 ± 0.0021 a	0.98 ± 0.04 a	132.34 ± 4.05 b	16.49 ± 0.2 a	92.5 ± 1.73 b
M	2.36 ± 0.11 a	4.27 ± 0.06 b	0.16 ± 0.06 a	80.26 ± 0.0018 a	0.99 ± 0.06 a	192.53 ± 7.87 a	15.21 ± 0.19 b	291 ± 1.73 a

Note: All data are presented as means ± standard deviation (SD). Different letters in the same column indicate significant differences among treatments at $p < 0.05$. SWC, soil water content; TN, total nitrogen content; TP, total P content; TK, total potassium content; AN, available nitrogen content; AP, available phosphorus content; AK, available potassium content. The same abbreviations are used in the tables below.

https://doi.org/10.1371/journal.pone.0278303.t002

the eggNOG database to obtain the descriptive information of each COG, as well as its functional information.

Statistical analyses

The trial data were statistically analyzed using Excel 2019 and Statistical Product and Service Solutions (SPSS) Statistics 21, and the results are shown as means with their standard deviations (mean ± SD). An online data analysis was performed using the free online Majorbio Cloud Platform (http://www.majorbio.com) of Majorbio Bio-Pharm Technology Co., Ltd. (Shanghai, China). The variance inflation factors (VIFs) of different sample environmental variables were calculated, and redundant variables were removed. The significance was based on 999 Monte Carlo permutations. A linear discriminant analysis (LDA) and a linear discriminant analysis effect size (LEfSe) method were used to identify significantly different bacterial and fungal communities under different environmental samples [48].

Results

Soil physicochemical properties

As shown in Table 2, the soil AP content and pH in the non-moss-growth area were significantly higher than those of the moss-growth area. On the contrary, the contents of soil AN and AK in moss-growth area were significantly higher than those of the non-moss-growth area. Meanwhile, the contents of soil TN, TP, TK and SWC were not significant difference between moss and non-moss growth areas ($p > 0.05$) (Table 2).

Soil microbial biomass

As seen in Table 3, only MBN in the moss-growth area was significantly higher than that of non-moss growth area. In contrast, MBC and MBP were not significantly different between moss and non-moss growth areas (Table 3).

Soil enzyme activities

β-Glucosidase, aminopeptidase, and phosphatase are closely related to soil C, N, and P cycles, respectively. The results of the enzyme activities in soils of the moss and non-moss-growth

Table 3. Soil microbial biomass C, N, and P between moss- (M) and non-moss growth(L) areas in litchi orchard ($mg{\cdot}kg^{-1}$).

Samples	Microbial Biomass C	Microbial Biomass N	Microbial Biomass P
L	139.2 ± 98.09 a	3.23 ± 2.98 b	30.08 ± 9.76 a
M	101.12 ± 50.5 a	11.29 ± 3.61 a	20.33 ± 15.27 a

Note: All data are presented as means ± standard deviation (SD). Different letters in the same column indicate significant differences among treatments at $p < 0.05$.

https://doi.org/10.1371/journal.pone.0278303.t003

Table 4. Soil enzyme activities related to C, N, and P cycles between moss (M) and non-moss-growth (L) areas in litchi orchard (nmol·g^{-1}·min^{-1} (30˚C)).

Samples	*β*-Glucosidase	Aminopeptidase	Acid phosphatase
L	0.6 ± 0.03 b	11.77 ± 0.81 a	0.97 ± 0.04 a
M	1.13 ± 0.13 a	6.77 ± 1.79 b	0.33 ± 0.26 a

Note: All data are presented as means ± standard deviation (SD). Different letters in the same column indicate significant differences among treatments at $p < 0.05$.

https://doi.org/10.1371/journal.pone.0278303.t004

areas in litchi orchard were as follows. The activity of *β*-glucosidase was significantly higher in soils of the moss-growth area than those of the non-moss-growth area. By contrast, the activity of aminopeptidase was significantly higher in soils of the non-moss-growth area than those of the moss-growth areas However, the activities acid phosphatase between moss and non-moss growth area were not significant differences between each other (Table 4).

Correlation between soil bacterial community and the environmental factors

Based on the variance inflation factor (VIF) analysis, the environmental factors with $p > 0.05$ or VIF > 20 were screened and removed. First, as the VIF values of available potassium (AK) and total phosphorus (TP) were greater than 20. Therefore, they were removed. And the remained environmental factors were used for a correlation heatmap analysis. The results showed that Proteobacteria and Acidobacteria were positively correlated with available phosphorus (AP) in soil, which indicated that higher contents of AP are suitable for their growth. Meanwhile, there was also a significant positive correlation between Cyanobacteria with the contents of total nitrogen (TN) and available nitrogen (AN). Moreover, the content of the total potassium (TK), it was positively and negatively related to Chloroflexi and Actinobacteria, respectively. Furthermore, Proteobacteria was positively correlated with pH And Cyanobacteria was positively correlated with SWC. A linear regression analysis also showed that TN (R^2 = 0.8231, p = 0.0125), TK (R^2 = 0.3, p = 0.2606), AP (R^2 = 0.0162, p = 0.8099), AN (R^2 = 0.1331, p = 0.4771), pH (R^2 = 0.1498, p = 0.4485), and SWC (R^2 = 0.2445, p = 0.3074) which suggested that soil bacterial community structure could be altered by the main environmental factors, particularly, the content of the total nitrogen was the most significant factor among the above environmental factors (Fig 2).

Alpha and *Beta* diversities of soil bacterial community

The Shannon and Simpson indices (Fig 3A and 3B), which describe soil bacterial diversity, did not show significant differences between moss and non-moss growth areas. Moreover, Ace and Chao1, the bacteria richness indices, also showed the same trends between moss and non-moss growth areas. These results suggested that soil bacterial diversity and richness were not significantly changed in moss growth areas which compared to the non-moss growth areas in litchi orchard (Fig 3C and 3D).

Based on the Bray–Curtis distance from PCoA, the bacterial community was significantly aggregated into two groups corresponding to the compositions of the soil bacterial communities between non-moss and moss-growth areas in litchi orchard (ANOSIM: R = 0.3333, p = 0.1990; Adonis: R^2 = 0.3336, p = 0.2000). At the OTU level, the contribution rates of the first principal component (PC1) and the second principal component (PC2) to the samples were 44.12% and 37.61%, respectively. Meanwhile, soil bacteria in the non-moss growth area grouped together which precisely separated with those of the moss growth area in litchi orchard (Fig 4).

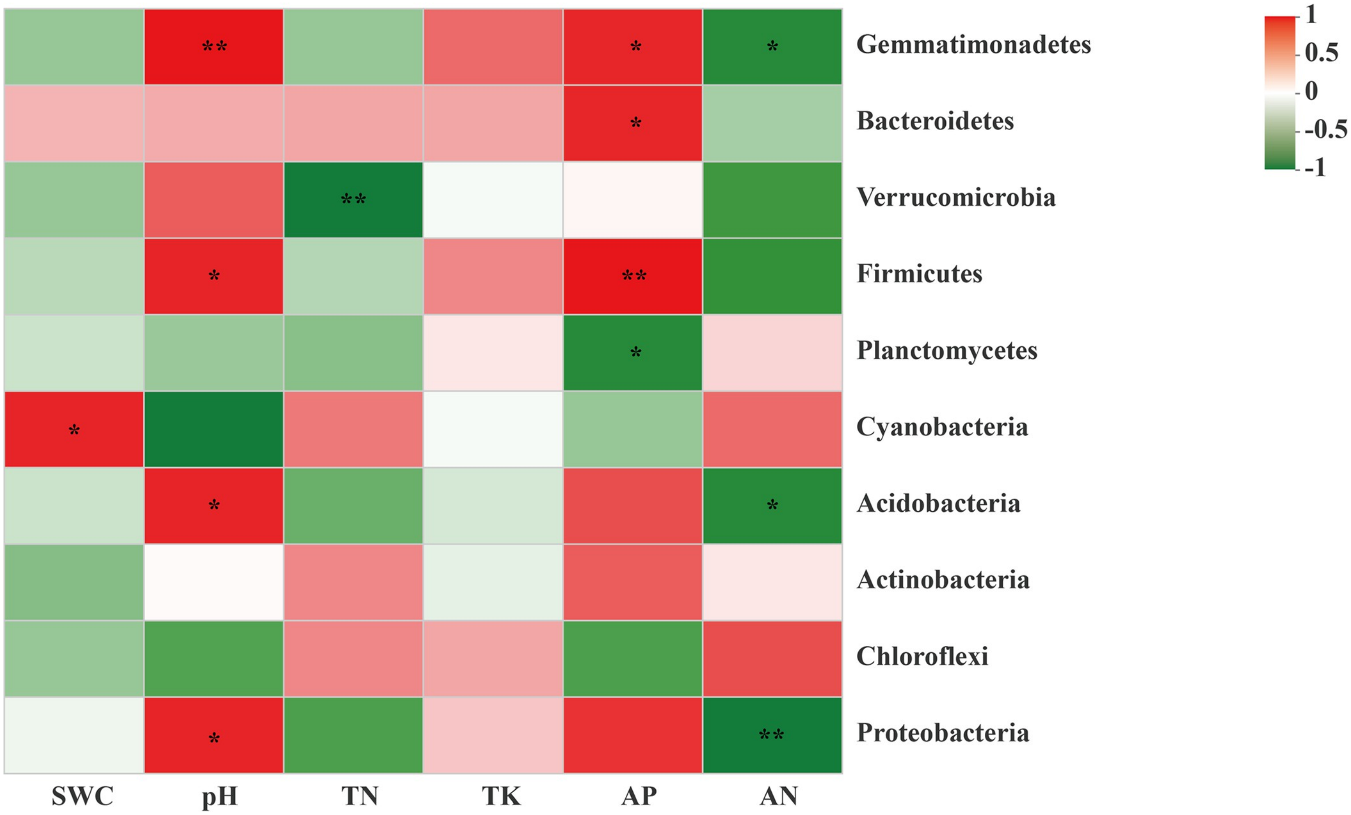

Fig 2. A correlation heatmap analysis between edaphic physicochemical factors and bacterial population at phylum level between moss- (M) and non-moss growth (L) areas in litchi orchard. *Note*: R values are shown in the figure in different shades of color, and they are marked with *, **, or ***, indicating *p* values less than 0.05, 0.01, and 0.001, respectively. SWC, soil water content; TN, total nitrogen; TK, total potassium; AN, available nitrogen; AP, available phosphorus.

https://doi.org/10.1371/journal.pone.0278303.g002

Compositions of soil microbial communities between moss and non-moss-growth areas in litchi orchard

At the phylum level, the numbers of soil-dominant bacterial phyla (i.e., relative abundances greater than 1%) in the moss and non-moss-growth areas in litchi orchard were 9 and 11, respectively. Firstly, the soil dominant soil bacterial phyla in the moss-growth area were Chloroflexi (33.51%), Actinobacteria (18.70%), Proteobacteria (18.18%), Cyanobacteria (10.38%), Acidobacteria (8.34%), Planctomycctcs (5.77%), WPS-2 (1.16%), Verrucomicrobia (1.02%), and others (1.65%), respectively (Fig 5A). In contrast, soil dominant bacterial phyla in non-moss-growth area were Proteobacteria (37.58%), Actinobacteria (19.22%), Acidobacteriota (15.15%), Chloroflexi (13.87%), Planctomycctcs (4.17%), Firmicutes (2.69%), Verrucomicrobia (1.65%), Bacteroidetes (1.34%), Gemmatimonadetes (1.23%) and others (2.27%), respectively. In comparison with the non-moss growth area, not only Chloroflexi enriched as the most abundant soil bacterial phylum in the moss-growth area, but also three soil-dominant bacterial phyla, namely, Firmicutes, Bacteroidetes, and Gemmatimonadetes lost in the moss-growth area. Meanwhile, WPS-2 was the soil dominant bacterial phylum in the moss growth area, but it was not the soil dominant bacterial phylum in the non-moss growth area. All above results suggested that soil bacterial compositions at the phylum level in moss growth areas were significantly different with those of the non-moss growth area in litchi orchard (Fig 5A).

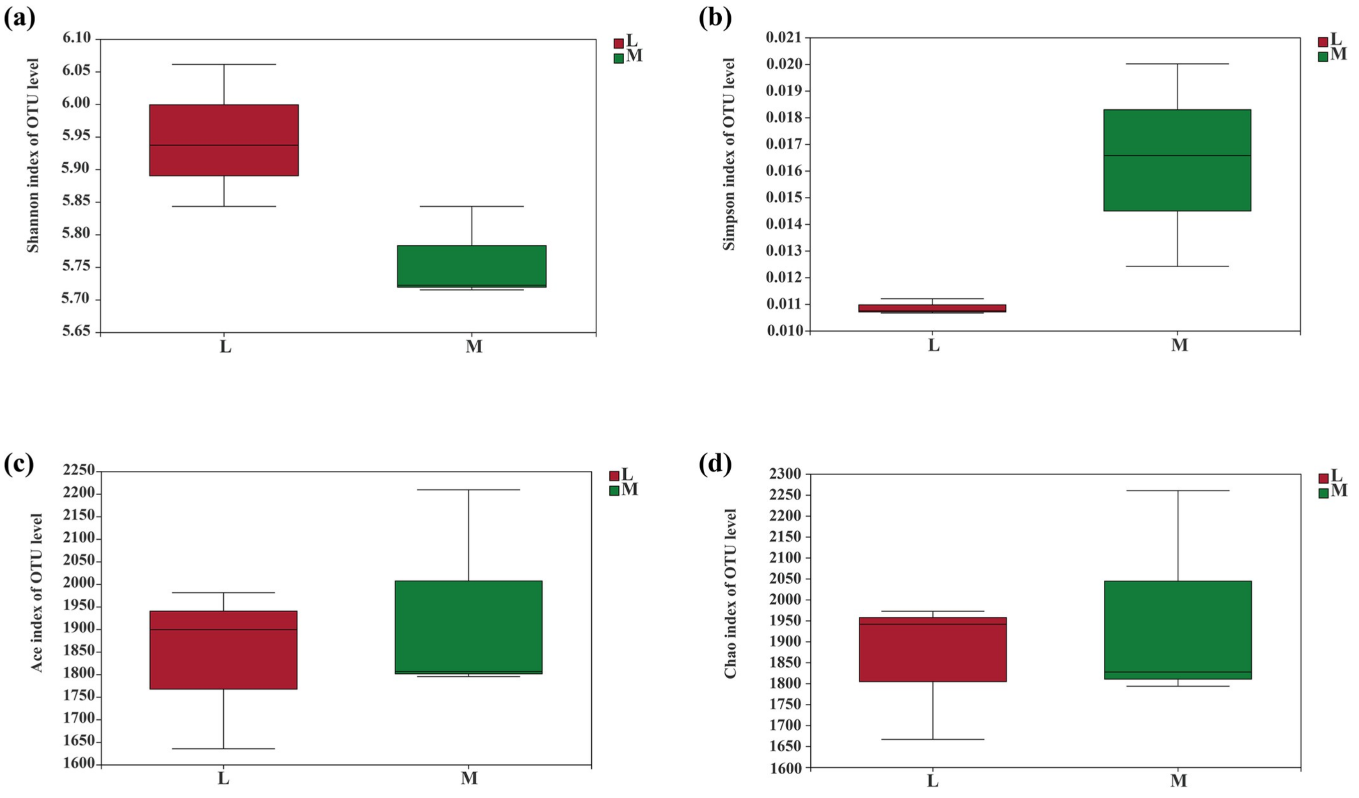

Fig 3. Soil bacterial diversity and richness between moss- (M) and non-moss growth (L) areas in litchi orchard: (a) Shannon index of OTU level. (b) Simpson index of OTU level. (c) Ace index of OTU level. (d) Chao index of OTU level.

https://doi.org/10.1371/journal.pone.0278303.g003

At the genus level, the numbers of soil-dominant bacterial genera (i.e., relative abundances greater than 1%) in the moss and non-moss growth areas in the litchi orchard were 22 and 17, respectively. The dominant soil bacterial genera in the moss-growth area were *norank_c_AD3* (12.52%), *norank_o_Chloroplast* (7.01%), *norank_f_Xanthobacteraceae* (6.24%), *Acidothermus* (5.71%), *norank_f_Gemmataceae* (4.50%), *HSB_OF53-F07* (3.88%), *unclassified_f_Ktedonobacteraceae* (2.90%), *norank_o_Acidobacteriales* (2.02%), *norank_o_Elsterales* (1.99%), *norank_c_TK10* (1.91%), *Bradyrhizobium* (1.85%), *Conexibacter* (1.64%), *norank_o_IMCC26256* (1.64%), *Bryobacter* (1.56%), *norank_o_SBR1-31* (1.42%), *norank_f_JG30-KF-AS9* (1.41%), *norank_o_B12-WMSP1* (1.27%), *Candidatus_Solibacter* (1.23%), *G12-WMSP1* (1.19%), *norank_p_WPS-2* (1.16%), *1921–2* (1.15%), *Pseudonocardia* (105%) and others (31.78%), respectively. By contrast, the soil dominant bacterial genera in the non-moss growth area were *norank_f_Xanthobacteraceae* (15.88%), *norank_c_AD3* (5.14%), *Acidothermus* (5.13%), *Candidatus_Solibacter* (3.25%), *norank_f_Gemmataceae* (2.94%), *norank_o_Subgroup_6* (2.89%), *Bradyrhizobium* (2.48%), *norank_o_IMCC26256* (2.22%), *norank_o_Acidobacteriales* (1.85%), *norank_o_Subgroup_2* (1.64%), *norank_o_Gaiellales* (1.60%), *norank_c_TK10* (1.57%), *Bryobacter* (1.57%), *HSB_OF53-F07* (1.37%), *norank_f_JG30-KF-AS9* (1.20%), *Candidatus_Koribacter* (1.15%), *Haliangium* (1.02%) and others (40.18%), respectively(Fig 5B).

Additionally, *Haliangium*, *Candidatus_Koribacter*, *norank_o_Gaiellales*, *norank_o_Subgroup_2*, and *norank_o_Subgroup_6* were the unique soil dominant bacterial genera in the non-moss growth area. In contrast, *1921–2*, *Pseudonocardia*, *norank_o_B12-WMSP1*, *norank_p_WPS-2*, *G12-WMSP1*, *norank_o_SBR1-31*, *Conexibacter*,

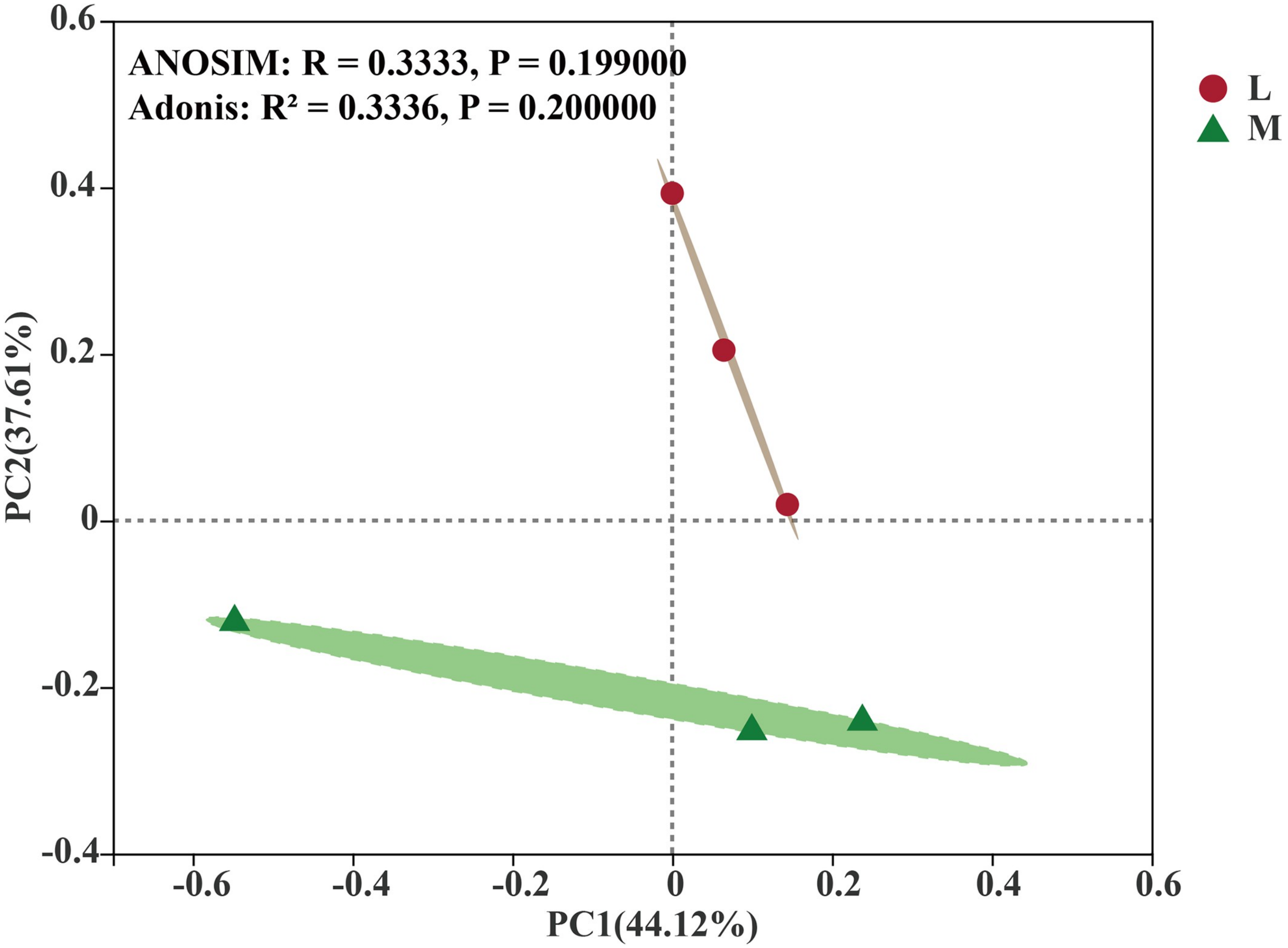

Fig 4. Principal coordinate analysis (PCoA) of soil bacterial communities between moss- and non-moss (L) growth (M) areas in litchi orchard based on Bray–Curtis distances.

https://doi.org/10.1371/journal.pone.0278303.g004

unclassified_f_Ktedonobacteraceae, *norank_o_Elsterales*, and *norank_o_Chloroplast* were the special soil dominant bacterial genera in the moss growth area.

At the genus level, 412 and 441 soil bacterial genera could be detected between the moss and non-moss-growth areas in the litchi orchard, respectively. Among them, there were 353 common soil bacterial genera between the moss and non-moss-growth areas. In addition, there were 59 and 88 unique soil bacterial genera in the moss-growth and non-moss-growth areas, respectively (Fig 6A). Moreover, at the OTU level, there were 2214 and 2376 soil bacterial genera in the moss and non-moss growth areas in the litchi orchard, respectively. Among them, 1727 common soil bacterial genera were found in the moss and non-moss growth areas. Moreover, there were 649 unique bacterial OTUs in the moss growth area and 487 unique bacterial OTUs in the non-moss-growth area (Fig 6B).

Significant differences of soil bacteria between the moss and non-moss growth areas in litchi orchard and the main contributing biomarker classes were examined using an LEfSe analysis (LDA threshold of 3.5) (Fig 7). A total of 51 soil bacteria clades showed statistically significant differences between moss and non-moss growth areas. Solirubrobacterales (from order to family), Leptolyngbyales (from order to family), Ktedonobacteria (from class to

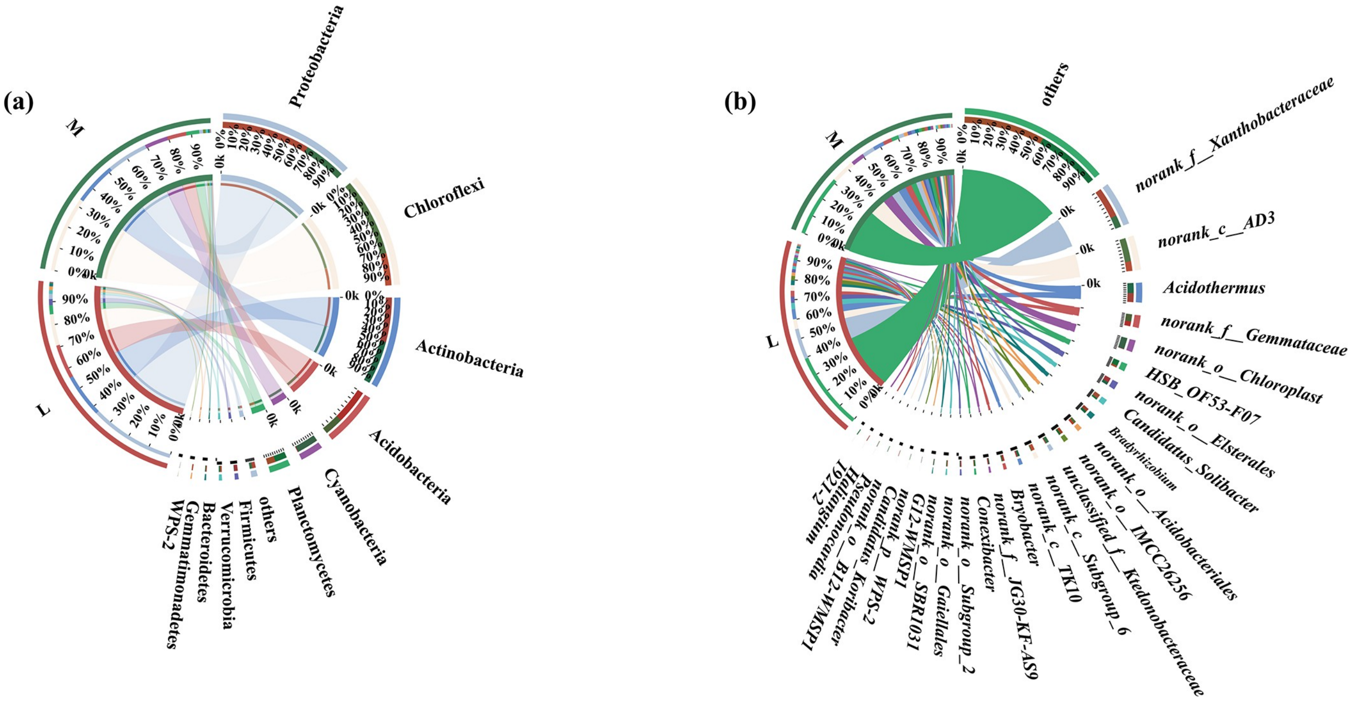

Fig 5. Distribution of soil-dominant bacteria at phylum level (a) and at genus level (b) between moss (M) and non-moss-growth (L) areas in litchi orchard.

https://doi.org/10.1371/journal.pone.0278303.g005

genus), norank_f__Ktedonobacteraceae (genus), 1921–2 (genus) and WPS-2 (from phylum to genus) enriched in the moss growth area.

In contrast, Firmicutes (from phyla to family), Acidobacteria (from phyla to genus), Candidatus_Solibacter (genus), Subgroup_6 (from class to genus), Proteobacteria (from phyla to genus), Alphaproteobacteria (class), Rhizobiales (order), Xanthobacteraceae (from family to genus), Hyphomicrobiaceae (from family to genus), Gammaproteobacteria (class), Betaproteobacteriales (from order to family), Aquaspirillaceae (from family to genus), norank_o__IMCC26256 (from family to genus), Leptolyngbyaceae (family), and Gemmatimonadetes (from phyla to genus) enriched in the non-moss growth area.

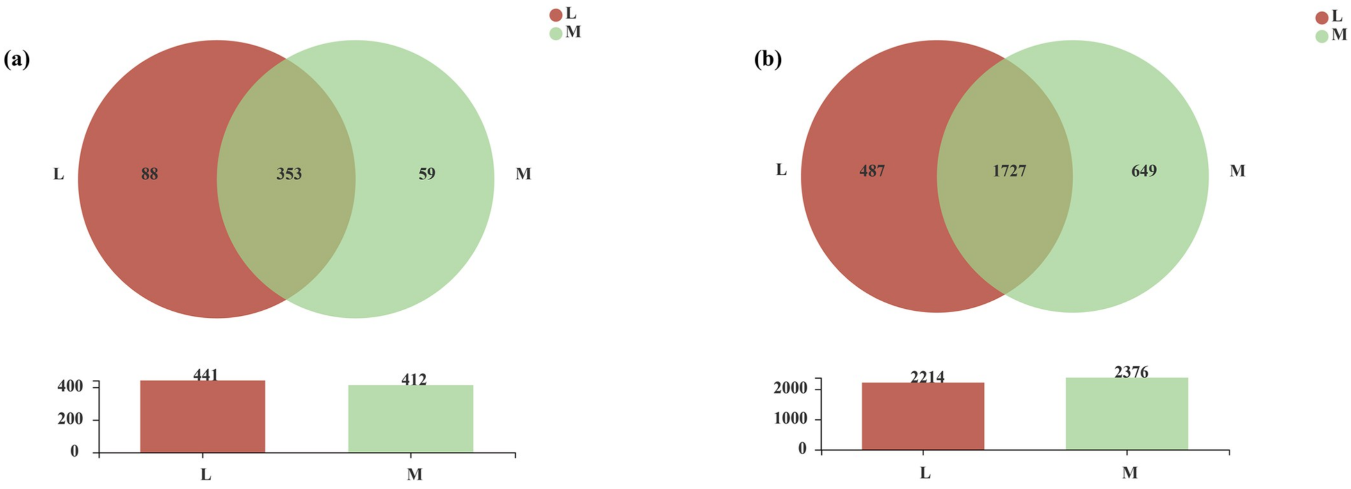

Fig 6. Venn analysis of soil bacteria between moss- (M) and non-moss growth (L) areas at the genus (a) and OTU (b) levels in litchi orchard.

https://doi.org/10.1371/journal.pone.0278303.g006

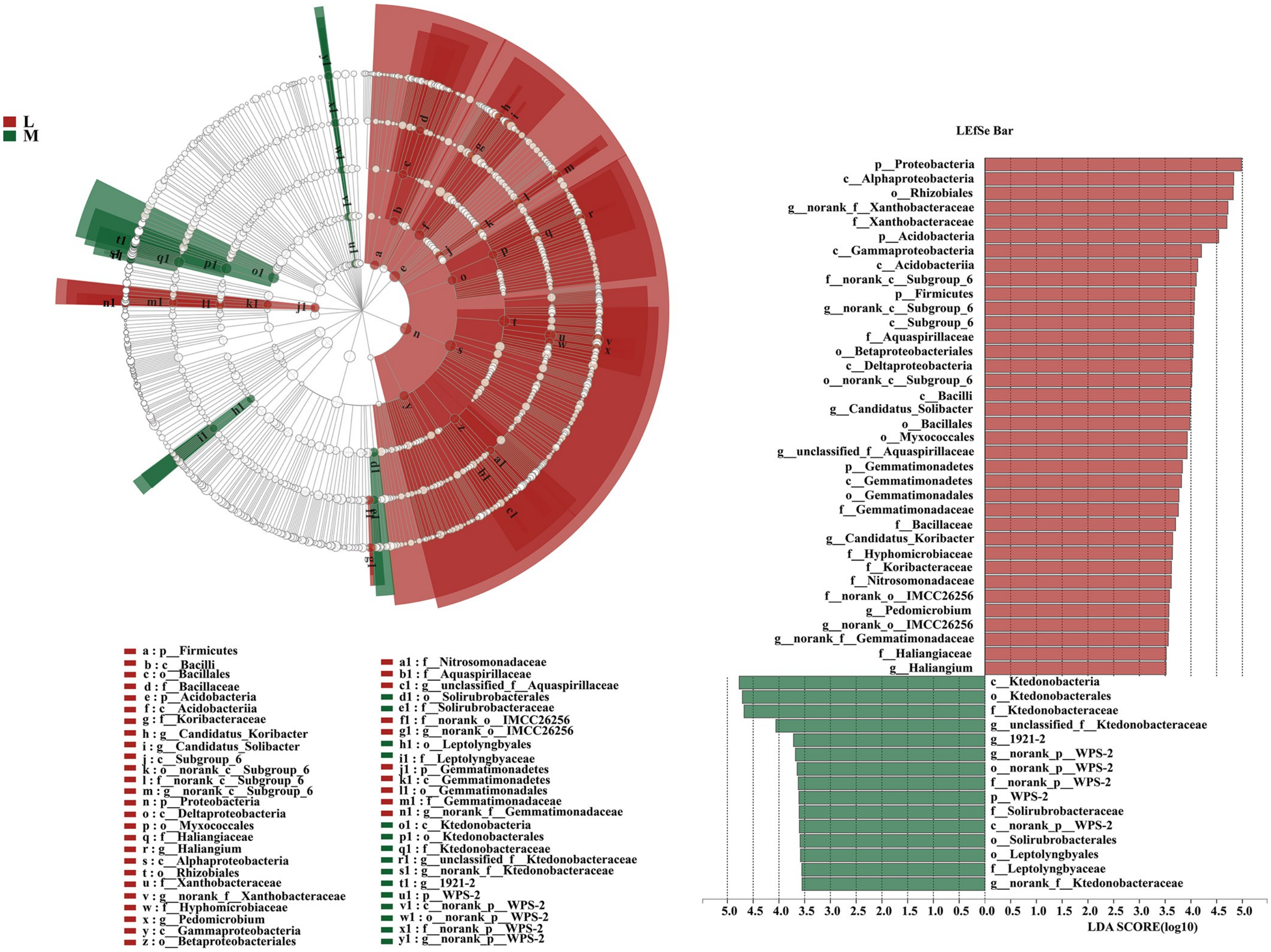

Fig 7. LEfSe analysis of significant marker groups of soil bacteria between non-moss (L) and moss-growth (M) areas in litchi orchard.

<https://doi.org/10.1371/journal.pone.0278303.g007>

Functional predictions of soil bacterial community structures

Based on the Kyoto Encyclopedia of Genes and Genomes (KEGG) database, the primary functional layer of soil bacteria in the moss and non-moss-growth areas contained six types of biometabolic pathways (Fig 8), i.e., Metabolism, Environmental Information Processing, Cellular Processes, Genetic Information Processing, Human Diseases, and Organismal Systems.

As seen in Fig 8, the percentages of the bio-functional pathways in the primary layer of the soil bacteria in the moss-growth area were inferior to those of the non-moss-growth area.

In addition, the secondary functional layer of soil bacterial genes in the moss and non-moss-growth areas mainly consisted of Membrane transport; Cellular community-eukaryotes; Signaling molecules and interaction; Circulatory system; Drug resistance: Antimicrobial; Cell motility; Antimicrobial eukaryotes; Signaling molecules and interaction; Circulatory system, etc. (Fig 9).

In Cell motility, Neurodegenerative disease, Infectious disease, viral, Cancer, specific types, Infectious disease, parasitic, Circulatory system and Cellular community-eukaryotes and others 40 secondary functional layers of soil bacteria in the moss growth area were degraded. However, the functional predictive gene types of Excretory system, Digestive system, Immune disease, Development and regeneration, Sensory system, and Signaling molecules and

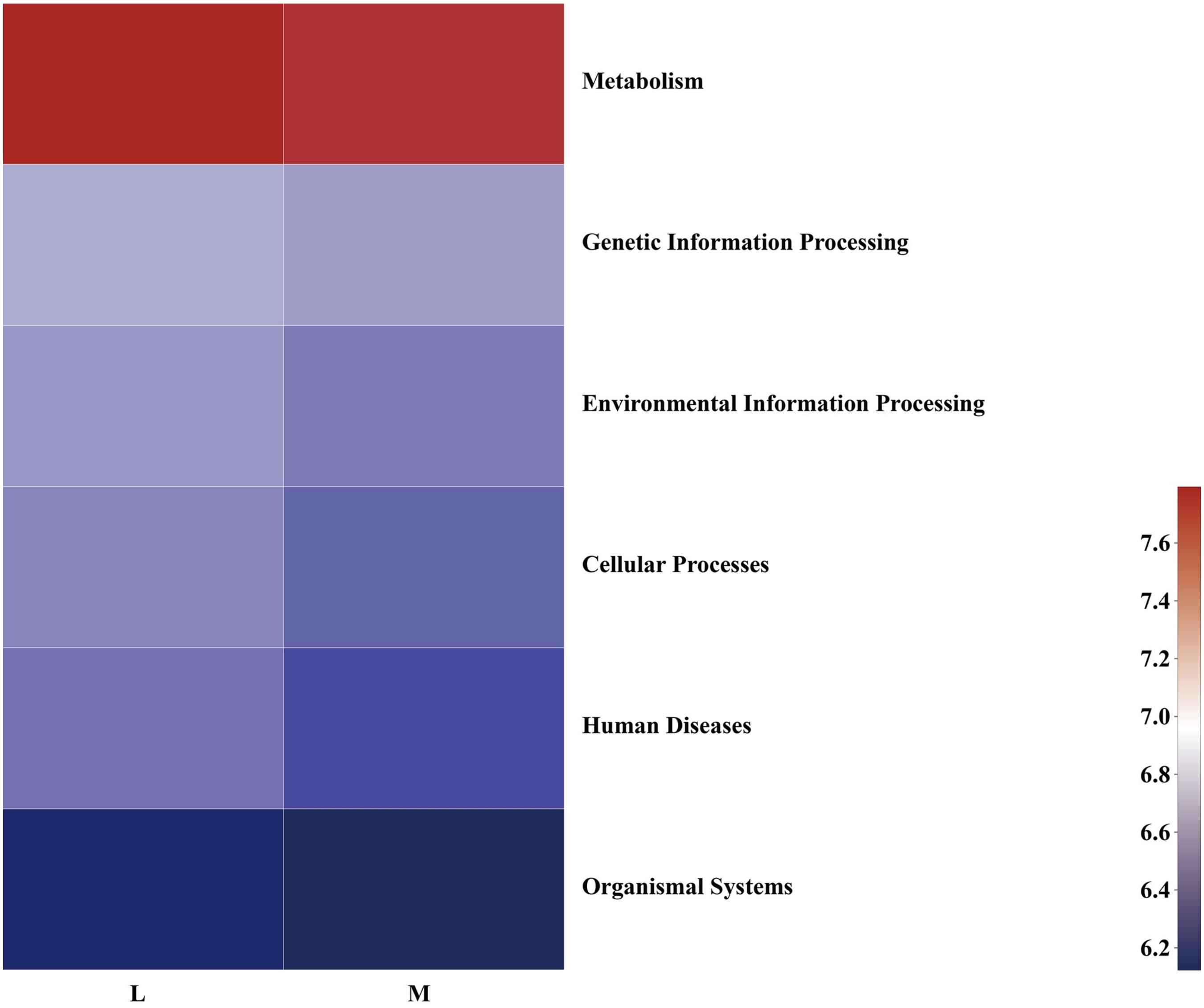

Fig 8. Variations in predicted soil bacterial functional profiles between moss (M) and non-moss growth(L) areas in litchi orchard (hierarchy level 1).

https://doi.org/10.1371/journal.pone.0278303.g008

interaction showed an increasing trend in the moss growth area. Particularly, in comparison with non-moss growth area, the Signaling molecules and interaction increased from 38.59% to 61.41% in the moss growth area. The above results indicated that although the predicted gene types in the secondary functional layer were similar between the moss and non-moss growth area, but the gene copy numbers were significant differences between each other. The functional degradation of soil bacteria in the moss growth area indicated that the functional abundances of soil bacterial genes at the secondary functional layer were lower than those of the non-moss growth area in the litchi orchard.

Correlation between soil fungal community and the environmental factors

The environmental factors with $p > 0.05$ or VIF > 20 were screened using a variance inflation factor (VIF) analysis. As the VIF values of the environmental factors, available potassium (AK) and total phosphorus (TP) were greater than 20, therefore, they were removed. Meanwhile,

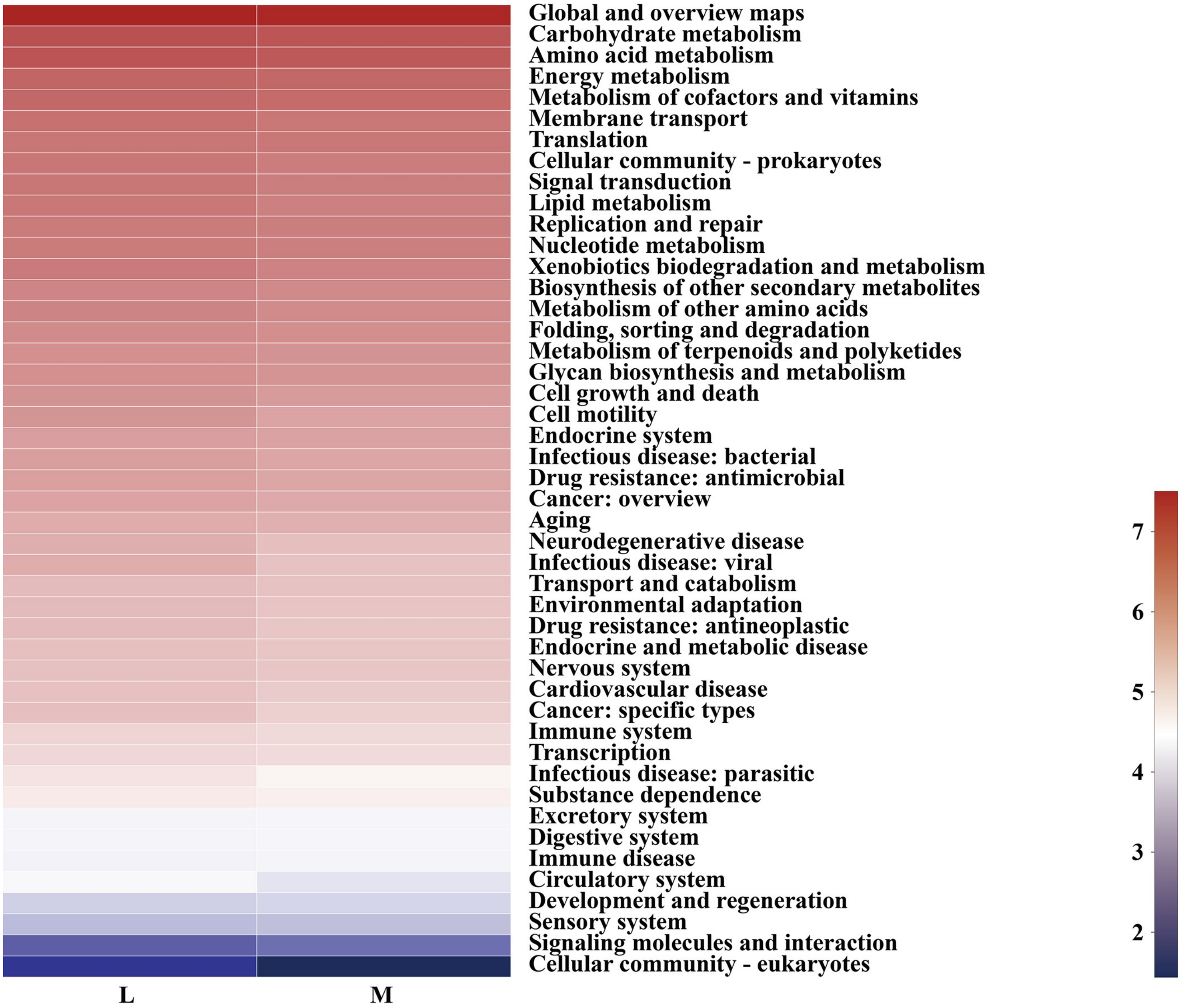

Fig 9. Variations in predicted functional profiles between moss- (M) and non-moss growth (L) areas in litchi orchard (hierarchy level 2).

https://doi.org/10.1371/journal.pone.0278303.g009

based on the correlation heatmap diagram analysis, soil pH, was a positive correlation with Mortierellomycota. Meanwhile, the contents of total potassium and available nitrogen were significantly negative correlation with unclassified_k__Fungi, Basidiomycota and Mortierellomycota. Furthermore, according to the linear regression analysis, TN ($R^2 = 0.4985$; $p = 0.1169$), TK ($R^2 = 0.002$; $p = 0.9323$), AP ($R^2 = 0.0162$; $p = 0.8099$), AN ($R^2 = 0.638$; $p = 0.0567$), pH ($R^2 = 0.6751$; $p = 0.0449$), and SWC ($R^2 = 0.2126$; $p = 0.3574$) were the mainly environmental factors in changing soil fungal communities, particularly, soil pH was the most significant factor among the above environmental factors ($p < 0.05$) (Fig 10).

Alpha and *Beta* diversities of soil fungal community

The Shannon and Simpson indices (Fig 11A and 11B) did not show significant differences in fungal diversities between moss and non-moss growth areas. Moreover, the Ace and Chao1

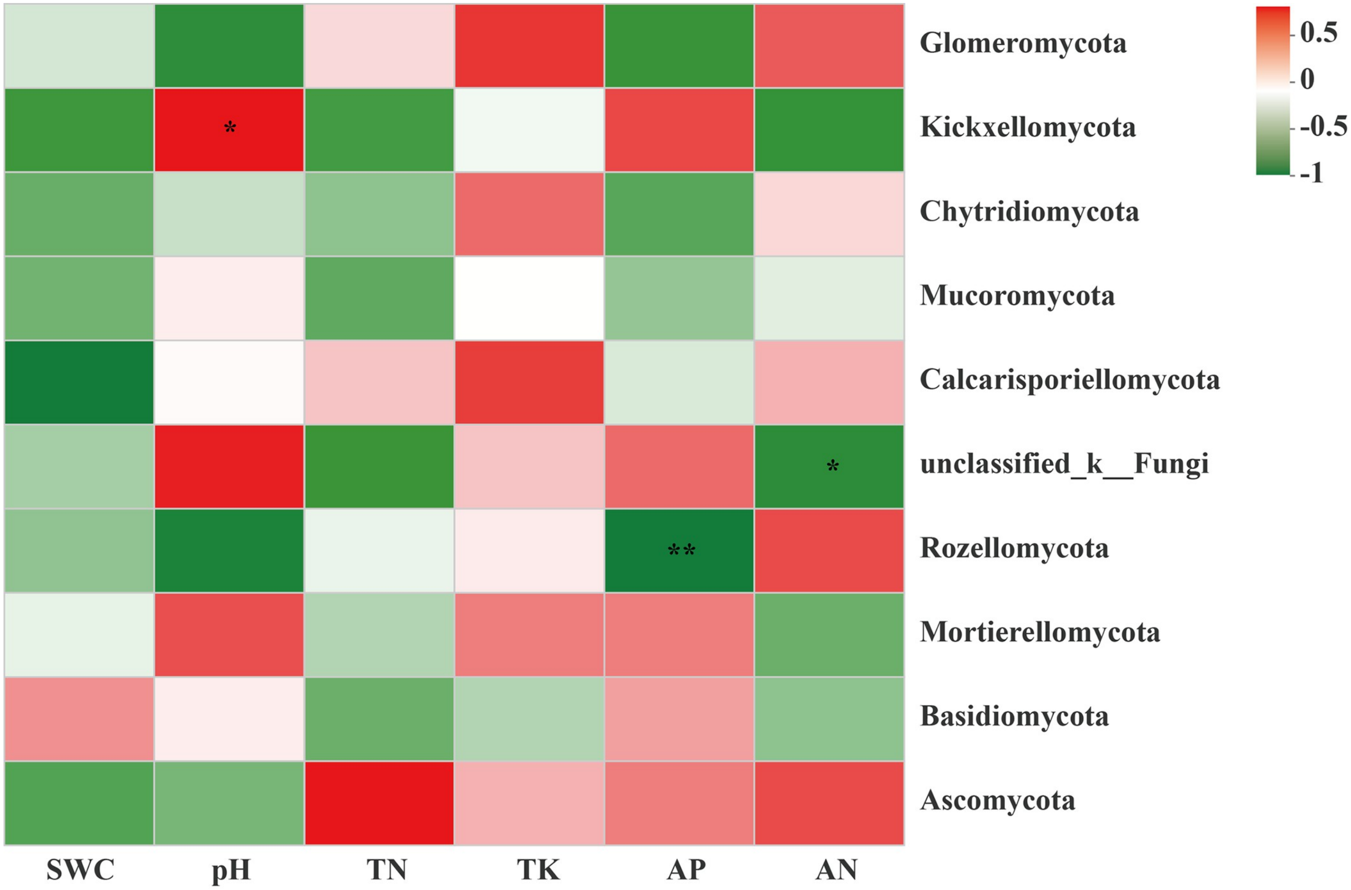

Fig 10. Correlation heatmap analysis between edaphic physicochemical factors and bacterial population at phylum level between moss- (M) and non-moss growth (L) areas in litchi orchard. *Note*: R values are shown in the figure in different shades of color, and they are marked with *, **, or ***, indicating *p* values less than 0.05, 0.01, and 0.001, respectively.

https://doi.org/10.1371/journal.pone.0278303.g010

indices, also showed the same trends with diversity between the moss and non-moss growth areas (Fig 11C and 11D). These results indicated that soil fungal diversity and richness in moss growth area did not significantly changed in litchi orchard.

Based on the Bray–Curtis distance from PCoA, the soil fungal communities were significantly clustered into two groups between moss and non-moss growth areas in litchi orchard (ANOSIM: R = 0.4815, p = 0.195; Adonis: R^2 = 0.3304, p = 0.100). As seen at Fig 12, the contribution rates of PC1 and PC2, as the first and the second principal components were 44.48% and 20.05%, respectively (Fig 12).

Compositions of soil fungal community between moss and non-moss-growth areas in litchi orchard

At the phylum level, five soil dominant fungal phyla (i.e., relative abundances greater than 1%) were detected between moss and non-moss growth areas in the litchi orchard. First, Ascomycota (56.99%), Basidiomycota (24.44%), Rozellomycota (9.09%), Mortierellomycota (8.46%) were the soil dominant fungal phyla of moss growth area in litchi orchard. By contrast, Ascomycota (54.45%), Basidiomycota (27.04%), Mortierellomycota (14.88%), unclassified_k_Fungi (4.17%) and others (0.81%) were the soil dominant fungal phyla of non-moss growth area in litchi orchard. In comparison with non-moss growth area, the proportions of Basidiomycota

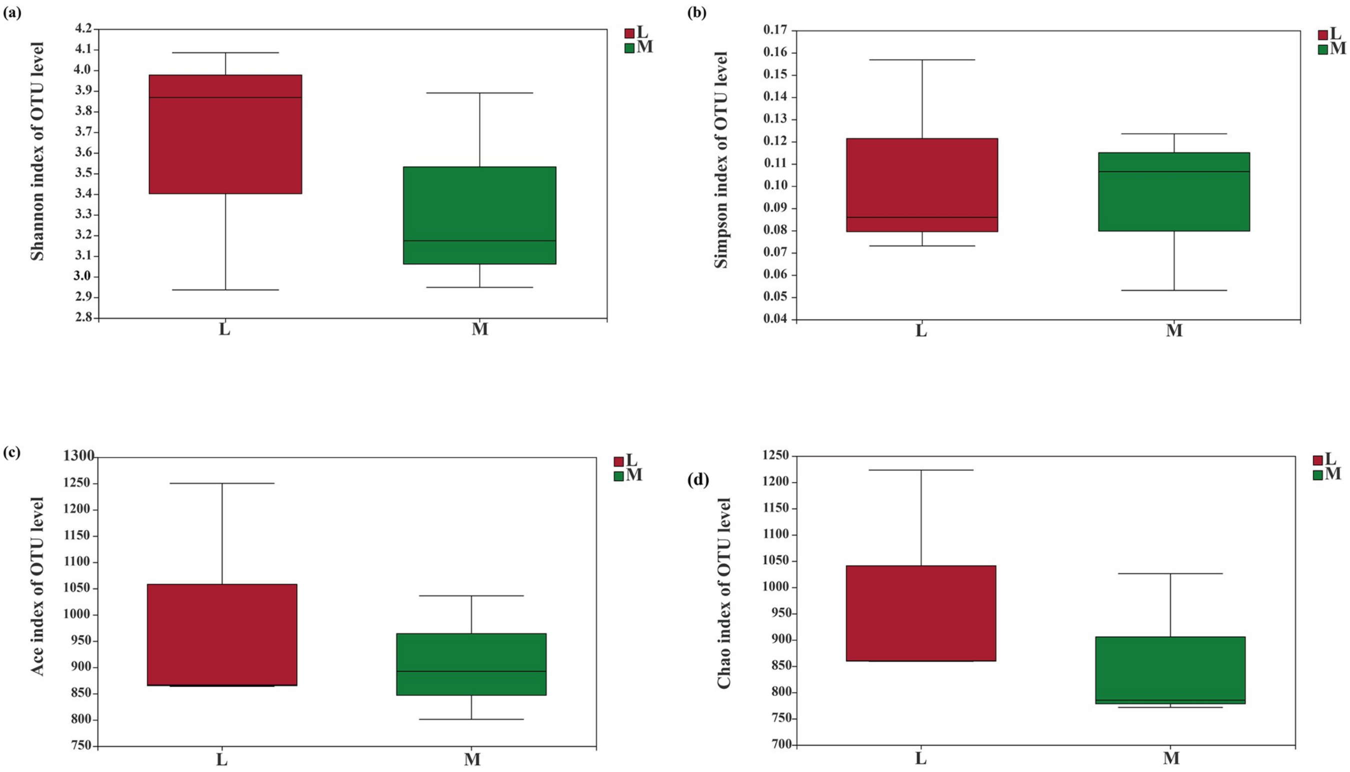

Fig 11. Soil fungal diversity and richness between moss- (M) and non-moss growth(L) areas in litchi orchard. *Note*: (a) Shannon index of OTU level. (b) Simpson index of OTU level. (c) Ace index of OTU level. (d) Chao index of OTU level.

https://doi.org/10.1371/journal.pone.0278303.g011

and Mortierellomycota decreased, but the proportion of Ascomycota increased in moss growth area. Moreover, unclassified_k_Fungi, as the soil dominant fungal phylum in the non-moss-growth area, but it lost in the moss growth area. Furthermore, Rozellomycota was the unique soil dominant fungal phylum of the moss growth area in litchi orchard (Fig 13A).

At the genus level, 14 soil-dominant fungal genera (i.e., relative abundances greater than 1%) were determined between moss and non-moss-growth areas in the litchi orchard. The proportions of soil dominant fungal genera in moss-growth area were ranked in order as follows: *unclassified_f_Thyridariaceae* (15.87%), *unclassified_p_Ascomycota* (12.09%), *Saitozyma* (9.19%), *unclassified_o_GS11* (8.68%), *Mortierella* (8.46%), *Rickenella* (6.72%), *Pyrenochaetopsis* (6.31%), *Penicillium* (3.52%), *Omphalina* (3.47%), *unclassified_f_Chaetomiaceae* (1.64%), *Tremella* (1.57%), *unclassified_c_Leotiomycetes* (1.29%), *Phoma* (1.17%), *Geminibasidium* (1.04%) and others (15.57%), respectively (Fig 13A). By contrast, in the non-moss-growth area, they were ranked as follows: *Saitozyma* (17.39%), *Mortierella* (14.20%), *Acaulium* (12.70%), *unclassified_p_Ascomycota* (10.77%), *Apiotrichum* (7.01%), *unclassified_k__Fungi* (4.17%), *Penicillium* (3.03%), *unclassified_c_Sordariomycetes* (2.58%), *unclassified_f_Thyridariaceae* (2.25%), *unclassified_f_Chaetomiaceae* (2.07%), *Trichoderma* (1.92%), *Fusarium* (1.46%), *unclassified_o_Hypocreales* (1.23%), *Pyrenochaetopsis* (1.22%) and others (16.7%), respectively (Fig 13B).

Meanwhile, unclassified_c_*Leotiomycetes*, *Phoma*, *Geminibasidium*, *Tremella*, *Omphalina*, *Rickenella*, and *unclassified_o_GS11t* were the unique soil dominant fungal genera in the moss growth area. In contrast, *unclassified_o_Hypocreales*, *Fusarium*, *Trichoderma*, *unclassified_c_Sordariomycetes*, *unclassified_k__Fungi*, *Apiotrichum* and *Acaulium* were the special soil dominant fungal genera in non-moss growth area in litchi orchard.

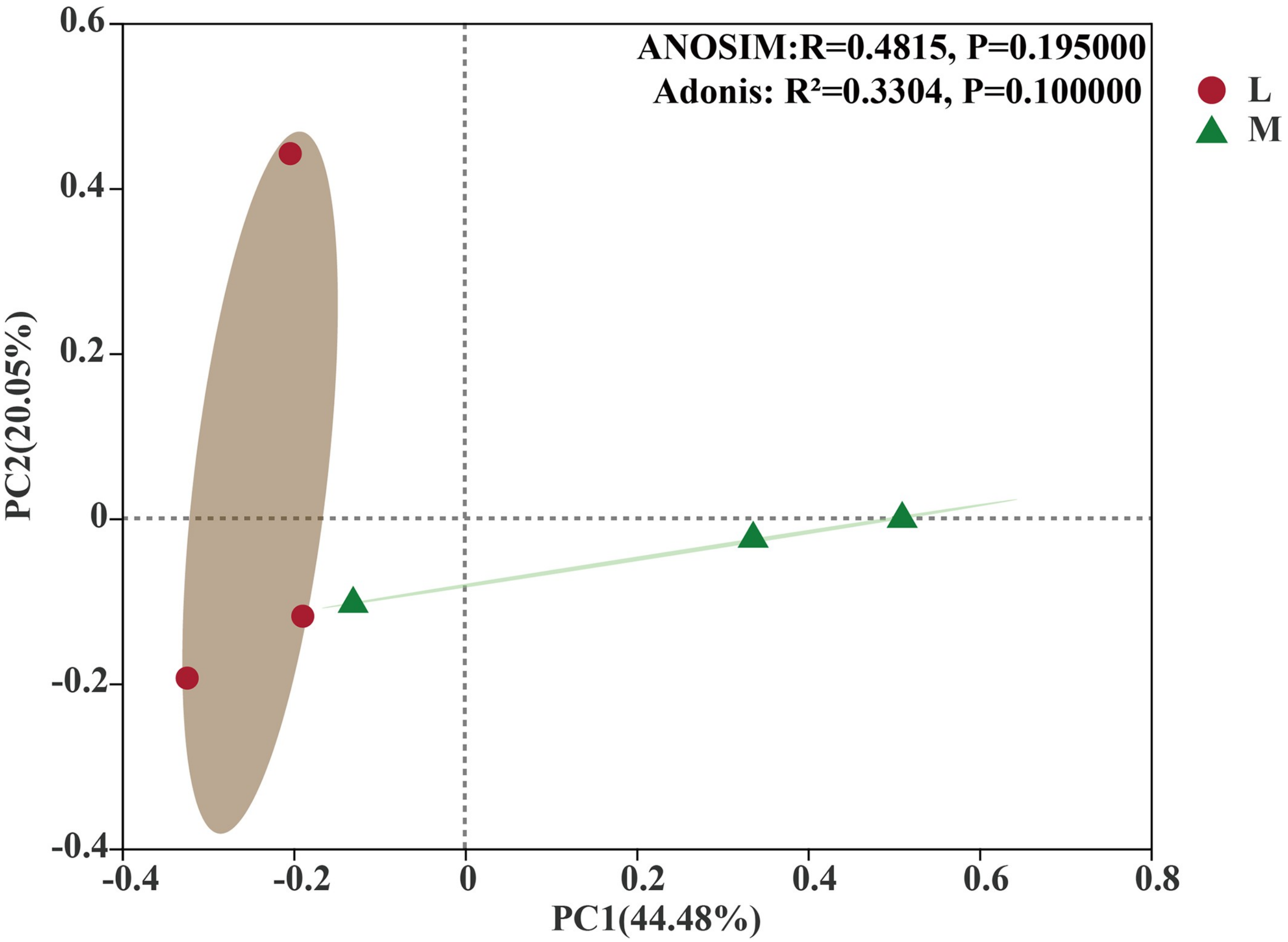

Fig 12. Principal coordinate analysis (PCoA) of fungal communities between moss- (M) and non-moss growth(L) areas in litchi orchard based on Bray–Curtis distances.

https://doi.org/10.1371/journal.pone.0278303.g012

Moreover, 302 and 337 soil fungal genera were obtained in the moss and non-moss growth areas in the litchi orchard, respectively. Among them, 238 common soil fungal genera were found between moss and non-moss-growth areas. Furthermore, 64 and 99 unique fungal genera were also detected in the moss and non-moss growth areas, respectively (Fig 14A). At the OTU level, 1238 and 1484 soil fungal OTUs were found in moss and non-moss growth areas, respectively. Among them, 790 OTUs were common fungal OTUs, and 448 and 694 were unique fungal OTUs in the moss and non-moss growth areas, respectively (Fig 14B). All above results suggested that soil fungal compositions had been changed in the moss-growth area. In comparison with the non-moss-growth area, not only at the genus level, but also at OTU levels, a degraded trend of the fungal compositions could be found in the moss-growth area in the litchi orchard.

Significant differences between moss and non-moss-growth areas in litchi orchard and the main contributing biomarker classes were examined using the LEfSe analysis (LDA threshold of 3.5) (Fig 15).

The compositions of the fungal communities differed significantly between the non-moss and moss-growth areas in litchi orchard according to the LEfSe analysis.

Glomerellales (from order to genus), Leotiomycetes (from class to genus), Thyridariaceae (from family to genus), Didymellaceae (from family to genus), Hymenochaetales (from order

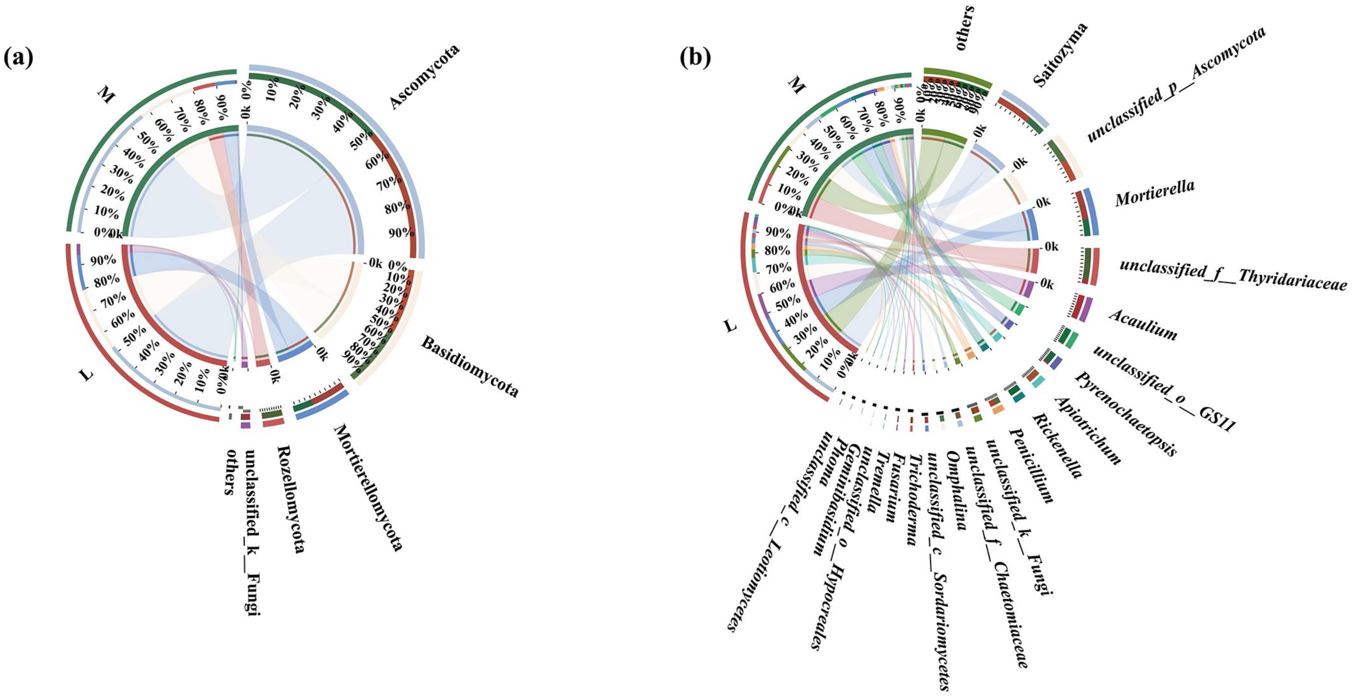

Fig 13. Distribution of soil-dominant fungi at phylum level (a) and at genus level (b) between moss- (M) and non-moss-growth (L) areas in litchi orchard.

https://doi.org/10.1371/journal.pone.0278303.g013

to genus), and Pucciniomycetes (from class to genus) enriched in soil of the moss-growth area. In contrast, Sordariomycetes (class), Nectriaceae (from family to genus), Microascales (from order to genus), and Onygenales (from order to genus) enriched in soil of the non-moss-growth area in litchi orchard.

Functional predictions of soil fungal community structures

By comparing with the EggNOG database, 16 COG functions were obtained. The analysis of interorganizational COG function composition showed that the top 10 COG functions in the

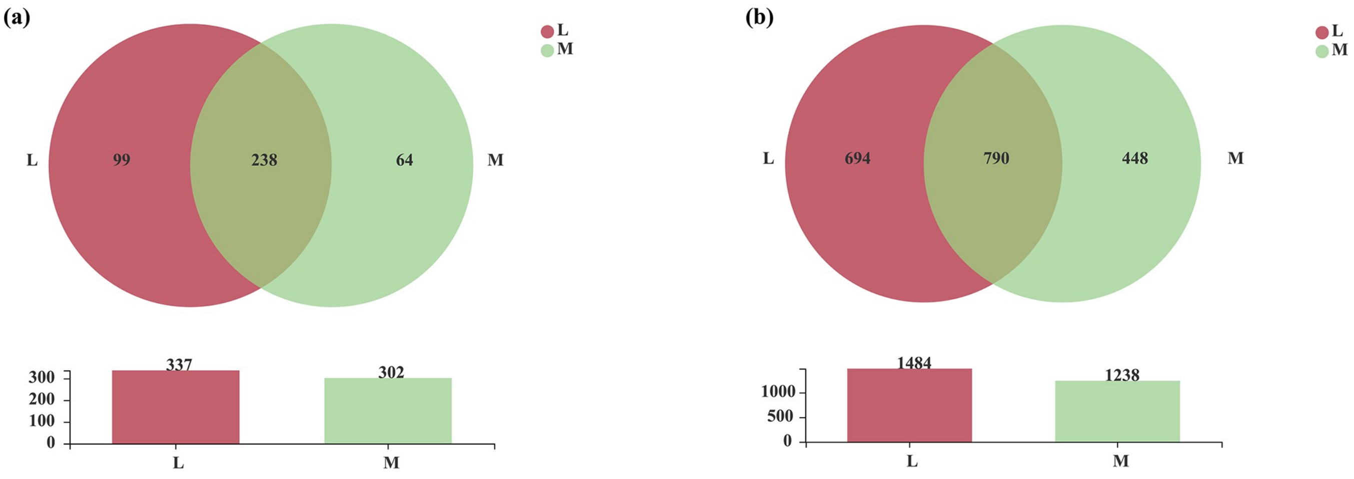

Fig 14. Venn analysis of soil fungi between moss- (M) and non-moss growth (L)areas at the genus (a) and OTU (b) levels in litchi orchard.

https://doi.org/10.1371/journal.pone.0278303.g014

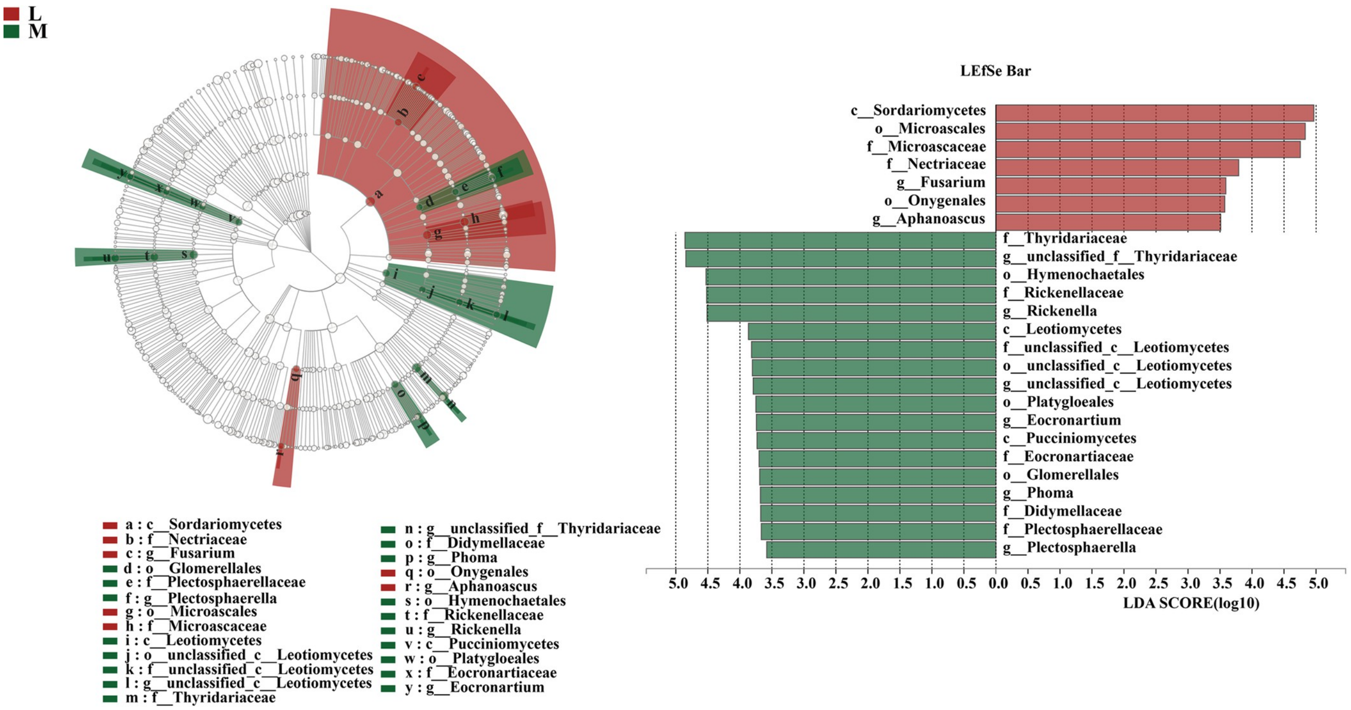

Fig 15. LEfSE analysis of significant marker groups of soil fungal communities between moss- (M) and non-moss growth(L) areas in litchi orchard.

https://doi.org/10.1371/journal.pone.0278303.g015

moss-growth area were Unknown (26.80%), Wood Saprotroph (16.09%), Undefined Saprotrop (10.03%), Fungal Parasite-Undefined Saprotroph (9.35%), Plant Pathogen (9.03%), Endophyte-Litter Saprotroph-Soil Saprotroph-Undefined Saprotroph (8.46%), Endophyte-Lichen Parasite-Undefined Saprotroph (6.31%), others (4.13%), Leaf Saprotroph (3.47%) and Soil Saprotroph (2.64%), respectively. In contrast, the top 10 COG functions in soil of the non-moss areas were Unknown (21.75%), Fungal Parasite-Undefined Saprotroph (17.52%), Endophyte-Litter Saprotroph-Soil Saprotroph-Undefined Saprotroph (14.27%), Animal Pathogen-Endophyte-Plant Pathogen-Undefined Saprotroph(12.88%), Undefined Saprotroph (11.78%), Soil Saprotroph (7.03%), others (4.76%), Animal Pathogen-Dung Saprotroph-Endophyte-Epiphyte-Plant Saprotroph-Wood Saprotroph (2.86%), Wood Saprotroph (2.48%), and Animal Pathogen-Endophyte-Lichen Parasite-Plant Pathogen-Soil Saprotroph-Wood Saprotroph (1.46%), respectively. Among them, the unique COG function of Animal fungicides-Endophyte-Plant fungicides-Undefined Saprotroph was only detected in the moss-growth area, and the special COG function of Leaf Saprotroph was detected in the non-moss-growth area. In the moss area, Wood Saprotroph, Endophyte-Lichen Parasite-Undefined, Saprotroph, Plant Pathogen, Endophyte-Dung Saprotroph-Lichen Parasite-Litter Saprotroph-Plant Pathogen-Soil Saprotroph-Wood Saprotroph and Fungal Parasite-Lichen Parasite Leaf Saprotroph, their functional taxa were higher than those of the non-moss-growth area (Fig 16).

Discussion

Soil fertility indexes between moss and non-moss growth areas in litchi orchard

Soil enzymes involved in the cycling of all nutrient elements, their activities related to the C, N, and P cycles in soil. And they had been regarded as the important biological indicators for soil

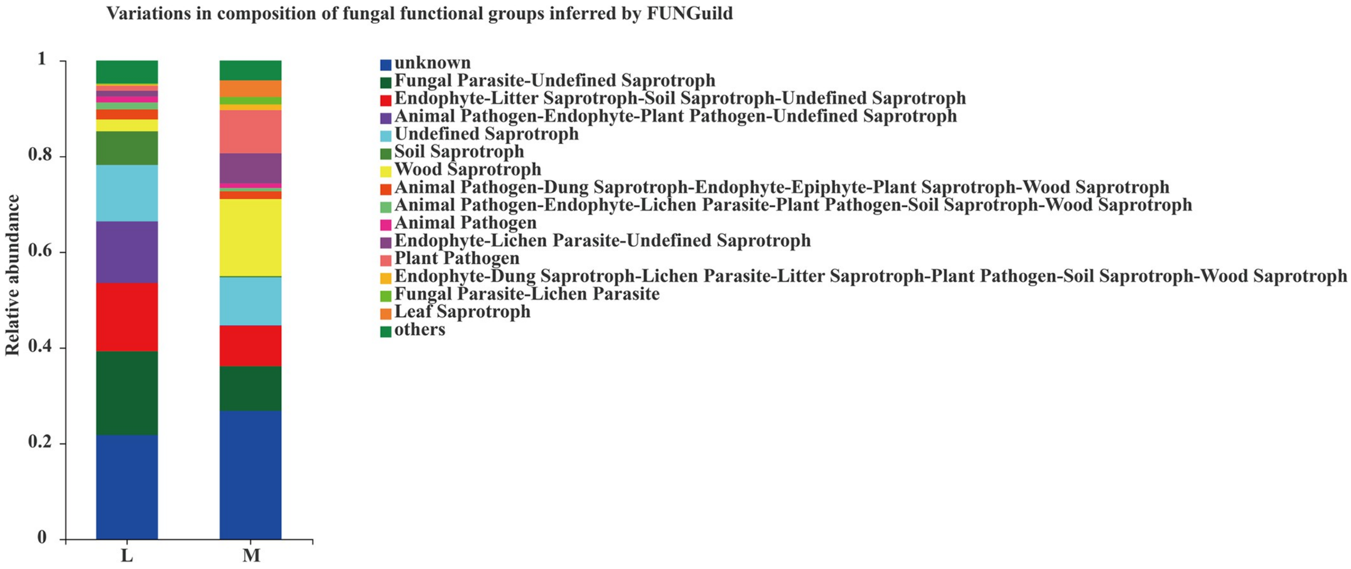

Fig 16. Relative abundance of soil fungal functional groups (guilds) based on OTU annotation table with distribution frequency level between moss- (M) and non-moss growth (L) areas in litchi orchard.

https://doi.org/10.1371/journal.pone.0278303.g016

fertility and ecological diversity, stability assessment [49–51]. Meanwhile, soil microbial biomass also can be used as the biological indicator for reflecting energy cycling, nutrient transport in soil [52, 53].

First, even though the contents of total nitrogen, phosphorus and potassium were not significantly different in soils between moss and non-moss growth areas. However, the contents of available nitrogen, phosphorus and potassium in soil were all significantly different between each other. Although the contents of available nitrogen and potassium in soil of moss growth area were significantly higher than those of non-growth area, but the content of available phosphorus significantly decreased in moss growth area. Meanwhile, in comparison with non-moss growth area, soil pH in moss growth area was also significantly declined.

In addition, MBC and MBP were not significantly different between moss and non-moss growth areas, but the MBN in moss growth area was significantly higher than that of non-moss-growth area. Moreover, except of the activity of acid phosphatase, the activities of β-Glucosidase and aminopeptidase in soils were all significantly different between moss and non-moss growth area. i.e., the activities of β-Glucosidase and aminopeptidase in soil of moss growth area were significantly higher and lower than those of non-moss growth area, respectively. All above results suggested that soil fertility in moss growth area exactly had already changed. Particularly, soil acidification and phosphorus deficiency were the soil primary features of the moss growth area in litchi orchard.

Soil microbial compositions and functions between moss and non-moss growth areas in litchi orchard

Soil microorganisms can be used as important indicators to evaluate soil fertility and represent its changes [54]. Numerous studies have confirmed that soil environmental factors have a significant influence on soil microbial community structure [55]. Soil nutrient status also can affect the functional diversity and abundance of microbial populations in soil ecosystems [56]. As proteobacteria and firmicutes are eutrophic bacteria, which grow rapidly under eutrophic conditions [57, 58]. However, the proportion of proteobacteria in soils of the moss growth

area was 18.18% only, but it was 37.58% in the non-moss growth area. Meanwhile, Firmicutes was not the soil dominant bacterial phylum in the moss growth area, but it was the soil dominant bacterial phylum in the non-moss-growth area. Moreover, as soil fertility can be significantly improved by Mortierellomycota, which it can promote the contents of soluble carbon, available phosphorous and phosphorus-related enzyme activities [45, 59]. And we also found that the proportion of Mortierellomycota in the non-moss growth area was also higher than that of the moss growth area. In addition, Chloroflexi and Cyanobacteria also are considered as the malnourished bacteria [60, 61]; WPS-2 generally lives in low soil fertility [62]; and Rozellomycota also shows strong adaptations to extreme soil environments, such as extreme pH levels and soil temperatures [63]. However, in comparison with non-moss growth area, Chloroflexi, Cyanobacteria, WPS-2 and Rozellomycota were all the soil dominant microbes in moss growth area. All above results indicated that higher nutrients status in non-moss growth area could be inferred than those of -moss growth area.

In addition, although soil bacteria and their metabolic pathways of the primary functional level between moss and non-moss growth areas were similar, but some bacterial gene functions, such as the bio-functional pathway, its proportion was lower in the moss growth area than that of the non-moss growth area. Furthermore, most of the fungal functional group's proportions in moss growth area were also found lower than those of the non-moss growth area. The results suggested that non only soil microbial compositions degraded, but also soil microbial functions also declined in moss growth areas which compared to non-moss growth areas in litchi orchard.

Conclusions

In comparison with the soils of the non-moss growth area, soil pH and the contents of AP were significantly decreased in the moss growth area. Meanwhile, the abundances of hypotrophic microorganisms, such as Chloroflexi, Cyanobacteria and WPS-2 enriched in the soils of the moss-grown area. Moreover, the proportions of eutrophic microorganisms, such as Proteobacteria and Firmicutes also declined and the metabolic pathways of soil microbial functional groups were also degraded in the moss growth area in litchi orchard. In one word, moss can be considered using as a visual bio-indicator for representing soil degradation in litchi orchards.

Author Contributions

Conceptualization: Siyu Chen.

Data curation: Siyu Chen.

Formal analysis: Siyu Chen.

Funding acquisition: Shandong Yang, Hongwei Tan.

Investigation: Siyu Chen.

Methodology: Siyu Chen, Yan Sun, Da Yang.

Project administration: Siyu Chen.

Resources: Shandong Yang.

Software: Yan Sun, Da Yang, Tian Liang.

Supervision: Shandong Yang.

Validation: Shandong Yang.

Visualization: Siyu Chen.

Writing – original draft: Siyu Chen.

Writing – review & editing: Siyu Chen, Shandong Yang.

References

1. Mitra SK. Overview of lychee production in the Asia-Pacific region. 2002.
2. Zhao L, Wang K, Wang K, Zhu J, Hu Z. Nutrient components health benefits and safety of litchi (*Litchi chinensis Sonn*): A review.Compr.Rev. Food Sci. Food Saf. 2020; 19 2139–2163. https://doi.org/10.1111/1541-433712590.
3. Stern RA, Adato I, Goren M, Eisenstein D, Gazit S. Effects of autumnal water stress on litchi flowering and yield in Israel.Sci.Hortic.1993; 54 295–302. https://doi.org/10.1016/0304-4238(93)90108-3
4. Huang XM, Li JG. Litchi production: History and current situation. 2007.
5. Menzel C M, Simpson D R. Lychee nutrition: A review. Sci.Hortic. 1987; 31 195–224. https://doi.org/10.1016/0304-4238(87)90046-X.
6. Guo-Liang LI, Zhang ZQ, Yao LX, Yang BM, Zhao HE, Zhou CM. Soil Nutrient Fertility in Litchi Orchards of Guangxi Zhuang Autonomous Region and Fujian Province. Chin. J. Soil Sci. 2012; 43 867–871.
7. Zheng YJ, Lin LW, Luo W. The nutrition characteristics of Litchi chinensis and the fertilizer practice. Soil Environ. Sci. 2001; volume pagination.
8. Fallahi E, Righetti TL, Raese JT. Ranking tissue mineral analyses to identify mineral limitations on quality in fruit.J.Am.Soc.Hortic.Sci.1988; 113 382–389.
9. Menzel C M, Haydon GF, Simpson DR. Mineral nutrient reserves in bearing litchi trees (*Litchi chinensis Sonn*). J. Hortic. Sci.1992; 67 149–160. https://doi.org/10.1080/00221589199211516232
10. Yurtseven E, Kesmez G D, Ünlükara A. The effects of water salinity and potassium levels on yield fruit quality and water consumption of a native central anatolian tomato species (*Lycopersicon esculantum*). Agric.Water Manag. 2005; 78 128–135. https://doi.org/10.1016/jagwat200504018
11. Pathak PK, Mitra SK. Rate and Time of Potassium Fertilization Influence Yield and Quality of Litchi. Acta Hortic. 2010; 863 235–242. https://doi.org/10.17660/actahortic2010 863 30.
12. Yao LX, Li GL, Yang BM, He ZH, Zhou CM, Tu S H. Effect of application ratio of potassium over nitrogen on litchi growth and fruit quality. Acta Hortic.2012; 1029 199–208. https://doi.org/10.17660/ActaHortic2014102923.
13. Weber BB, Belnap J. Biological Soil Crusts: An Organizing Principle in Drylands; Springer: Berlin/Heidelberg Germany. 2016. https://doi.org/10.1007/978-3-319-30214-0_1
14. Klenk N.Controls on Nutrient Availability in Black Spruce Forests of Northwestern Quebec.Master's Thesis McGill University Montreal QC Canada 2001
15. Yan D, Huang H, Zhang S, Xue B.Nutrients and particle composition characteristics in moss biological crusts.J.Arid Land Resour.Environ.2018 volume pagination
16. Badacsonyi A. Effects of Desiccation on Phosphorus and Potassium Acquisition by a Desiccation-tolerant Moss and Lichen.Ann.Bot.2000; 86:621–627. https://doi.org/10.1006/anbo20001228
17. Hao Z, Ji YE, Jiang P, Lin F. Roles of bryophyte in nutrient cycling in dark coniferous forest of Changbai Mountains.Chin.J.Appl.Ecol.2005; 16:2263–2266. https://doi.org/10.1360/aps040037
18. Kidron GJ. Do mosses serve as sink for rain in the Negev Desert? A theoretical and experimental approach.CATENA. 2014;12131–39. https://doi.org/10.1016/jcatena201405001
19. Zotz G, Schweikert A, Jetz W, Westerman H. Water relations and carbon gain are closely related to cushion size in the moss Grimmia pulvinata. New Phytol.2000; 148, 59–67. https://doi.org/10.1046/j.1469-8137.2000.00745.x PMID: 33863032
20. Cameron A J, Nickless G. Use of msses as collectors of airborne heavy metals near a smelting complex. Water. Air. Soil Pollut.1977; 7, 117–125. https://doi.org/10.1007/BF00283805
21. Longton RE. Studies on Growth Reproduction and Population Ecology in Relation to Microclimate in the Bipolar Moss Polytrichum alpestre. Bryologis. 1979; 82,325–367. https://doi.org/10.2307/3242212
22. Lee J, Johnson-Green P, Lee EJ. Correlation between Environmental Conditions and the Distribution of Mosses Exposed to Urban Air Pollutants. Water Air Soil Pollut. 2004;153,293–305. https://doi.org/10.1023/B%3AWATE000001994995159fa
23. Frego K A. Bryophytes as potential indicators of forest integrity. For. Ecol. Manag. 2007; 242:65–75. https://doi.org/10.1016/jforeco200701030.

24. Rillig MC, Mummey DL. Mycorrhizas and soil structure. New Phytol. 2010; 171:41–53. https://doi.org/10.1111/j1469–8137200601750x

25. Bardgett RD, Freeman C, Ostle N J.Microbial contributions to climate change through carbon cycle feedbacks.ISME J.2008; 2: 805–814. https://doi.org/10.1038/ismej200858

26. Evans RD. Microbiotic Crusts and Ecosystem Processes. Crit. Rev. Plant.Sci. 1999; 18:183–225. https://doi.org/10.1016/S0735-2689(99)00384-6

27. Anderson IC, Campbell CD, Prosser JI. Potential bias of fungal 18S rDNA and internal transcribed spacer polymerase chain reaction primers for estimating fungal biodiversity in soil. Environ. Microbiol. 2003; 5:36–47. https://doi.org/10.1046/j.1462-2920.2003.00383.x PMID: 12542711

28. Kaiser K, Wemheuer B, Korolkow V, Wemheuer F, Nacke H, Sch?Ning I, et al. Driving forces of soil bacterial community structure diversity and function in temperate grasslands and forests.Sci.Rep. 2016; 6 33696. https://doi.org/10.1038/srep33696 PMID: 27650273

29. Sun X, Zhou Y, Tan Y, Wu Z, Lu P, Zhang G, et al. Restoration with pioneer plants changes soil properties and remodels the diversity and structure of bacterial communities in rhizosphere and bulk soil of copper mine tailings in Jiangxi Province China.Environ.Sci.Pollut.Res.2018; 25:22106–22119. https://doi.org/10.1007/s11356-018-2244-3

30. Muhammad N, Dai Z, Xiao K, Meng J, Brookes P C, Liu X, et al. Changes in microbial community structure due to biochars generated from different feedstocks and their relationships with soil chemical properties. Geoderma. 2014;226–227,270–278. https://doi.org/10.1016/jgeoderma201401023

31. Sánchez-Cañizares C, Jorrín B, Poole P S, Tkacz A. Understanding the holobiont: The interdependence of plants and their microbiome. Curr.Opin. Microbiol.2017; 38:188–196 https://doi.org/10.1016/j.mib.2017.07.001 PMID: 28732267

32. Wu Y, Zeng J, Zhu Q, Zhang Z, Lin X. pH is the primary determinant of the bacterial community structure in agricultural soils impacted by polycyclic aromatic hydrocarbon pollution. Sci. Rep. 2017; 7:40093. https://doi.org/10.1038/srep40093 PMID: 28051171

33. Cui Y, Fang L, Guo X, Wang X, Zhang Y, Li P, et al. Ecoenzymatic stoichiometry and microbial nutrient limitation in rhizosphere soil in the arid area of the northern Loess Plateau China. Soil Biol. Biochem.2018; 116:11–21. https://doi.org/10.1016/jsoilbio201709025

34. Ye Z, Li J, Wang J, Zhang C, Dong Q. Diversity and co-occurrence network modularization of bacterial communities determine soil fertility and crop yields in arid fertigation agroecosystems. Biol. Fertil. Soils.2021; 57: 809–824 https://doi.org/10.1007/s00374-021-01571-3

35. Wei C, Yu Q, Bai E, Lü X, Li Q, Xia J, et al. Nitrogen deposition weakens plant-microbe interactions in grassland ecosystems. Glob. Change Biol.2013; 19:3688–3697. https://doi.org/10.1111/gcb.12348 PMID: 23925948

36. Powlson DS, Prookes PC, Christensen B T. Measurement of soil microbial biomass provides an early indication of changes in total soil organic matter due to straw incorporation. Soil Biol.Biochem.1987; 19:159–164. https://doi.org/10.1016/0038-0717(87)90076-9

37. Tabatabai M. Enzymes in soil: Research and developments in measuring activities. In Enzymes in the Environment: Activity Ecology and Applications; CRC Press: Boca Raton FL USA. 2002.

38. Lan X, Zhou H, Yao T, DongWQ, Zhang JG, Han DR. Community structure and function of cultivable Endo-phytic Bacteria isolated from four Moss species in Qilian Mountain. Symbiosis 80, 257–267 (2020). https://doi.org/10.1007/s13199-020-00669-w

39. Alvarenga DO, Kathrin R. Unraveling host–microbe interactions and ecosystem functions in moss–bacteria symbioses. Journal of Experimental Botany, 2022(13):13. https://doi.org/10.1093/jxb/erac091 PMID: 35728619

40. Menefee SG, Overman ORA Semimicro-Kjeldahl. Method for the Determination of Total Nitrogen in Milk.J. Dairy Sci.1940; 23:1177–1185. https://doi.org/10.3168/jdss0022-0302(40)92829-6

41. Xu YX, Du A, Wang ZC, Zhu WK, Li C, Wu LC. Effects of different rotation periods of Eucalyptus plantations on soil physiochemical properties, enzyme activities, microbial biomass and microbial community structure and diversity. Forest Ecology and Management, 2020; 456:117683. https://doi.org/10.1016/jforeco2019117683

42. Smith FW, Ellis BG, Grava J. Use of Acid-Fluoride Solutions for the Extraction of Available Phosphorus in Calcareous Soils and in Soils to Which Rock Phosphate Has Been Added1.Soil Sci.Soc.Am.J.1957; 21:400–404. https://doi.org/10.2136/sssaj19570361599500210004001 2x

43. Vance ED, Brookes PC, Jenkinson DS. An extraction method for measuring soil microbial biomass C. Soil Biol.Biochem.1987; 19:703–707. https://doi.org/10.1016/0038-0717(87)90052-6

44. Joergensen RG, Brookes PC. Ninhydrin-reactive nitrogen measurements of microbial biomass in 0 5 M K_2SO_4 soil extracts. Soil Biol.Biochem.1990; 22:1023–1027. https://doi.org/10.1016/0038-0717(90)90027-w

45. Hayano K. A method for the determination of β-glucosidase activity in soil.Soil Sci.Plant Nutr.1973; 19:103–108. https://doi.org/10.1080/00380768197310432524

46. Ladd JN. Properties of proteolytic enzymes extracted from soil. Soil Biol.Biochem.1972; 4:227–237. https://doi.org/10.1016/0038-0717(72)90015-6

47. Tabatabai MA, Bottomley PS, Angle JS, Weaver RW (Eds.), Methods of Soil Analysis: Part 2-microbiological and Biochemical Properties, Soil Science Society of America, Madison. (1994)

48. Segata N, Izard J, Waldron L, Gevers D. Metagenomic biomarker discovery and explanation. Genome Biol.2011 12 R60. https://doi.org/10.1186/gb-2011-12-6-r60 PMID: 21702898

49. Ndiaye EL, Sandeno JM, Mcgrath D, Dick R P. Integrative biological indicators for detecting change in soil quality.Am.J.Altern.Agric.2000; 15: 26–36. https://doi.org/10.1017/S0889189300008432

50. Sowerby A, Emmett B, Beier C, Tietema A, Pe?Uelas J, Estiarte M, et al. Microbial community changes in heathland soil communities along a geographical gradient: Interaction with climate change manipulations. Soil Biol.Biochem.2005; 37:1805–1813. https://doi.org/10.1016/jsoilbio200502023

51. Bocar A, Huang Q, Chen W, Wen S, Zhang J, Ibrahim M, et al. Microcalorimetric assessment of microbial activity in long-term fertilization experimental soils of Southern China. Fems Microbiol. Ecol. 2010; 70:30–39. https://doi.org/10.1111/j1574–6941200900753x.

52. Coyne MS. Soil Microbiology Ecology and Biochemistry 3rd Edition. Vadose Zone J. 2009; 8:1087–1088. https://doi.org/102136/vzj20090053br

53. Doran Coleman DC, Bezdicek DF.Defining Soil Quality for a Sustainable Environment; John Wiley & Sons: Hoboken NJ USA.1994.

54. Liu J, Liu W, Long XE, Chen Y, Huang T, Huo J, et al. Effects of nitrogen addition on C:N:P stoichiometry in moss crust-soil continuum in the N-limited Gurbantünggüt Desert Northwest China. Eur. J. Soil Biol. 2020; 98:103174. https://doi.org/10.1016/jejsobi2020103174

55. Sharma SK, Ramesh A, Sharma MP, Joshi OP, Karlen DL. Microbial Community Structure and Diversity as Indicators for Evaluating Soil Quality. In Biodiversity Biofuels Agroforestry and Conservation Agriculture; Springer: Dordrecht The Netherlands. 2010. https://doi.org/10.1007/978-90-481-9513-8_11

56. Wang Z, Zhang Q, Staley C, Gao H, Ishii S, Wei X, et al. Impact of long-term grazing exclusion on soil microbial community composition and nutrient availability. Biol. Fertil. Soils. 2019; 55:121–134. https://doi.org/10.1007/s00374-018-01336-5

57. Tiedje JM, Cho JC, Murray A, Treves D, Zhou J. Soil teeming with life: New frontiers for soil science. 2001. https://doi.org/10.1079/97808519946590393

58. Smit E, Leeflang P, Gommans S, Broek JVD, Wernars K. Diversity and Seasonal Fluctuations of the Dominant Members of the Bacterial Soil Community in a Wheat Field as Determined by Cultivation and Molecular Methods. Appl. Environ.Microbiol. 2001; 67:2284–2291. https://doi.org/10.1128/AEM.67.5.2284-2291.2001 PMID: 11319113

59. Fierer N, Bradford MA, Jackson RB.Toward an Ecological Classification of Soil Bacteria.Ecology. 2007; 88:1354–1364. https://doi.org/10.1890/05-1839 PMID: 17601128

60. Sun R, Zhang P, Riggins CW, Zabaloy MC, Rodríguez-Zas S, Villamil MB. Long-Term N Fertilization Decreased Diversity and Altered the Composition of Soil Bacterial and Archaeal Communities.Agronomy. 2019; 9: 574. https://doi.org/10.3390/agronomy9100574

61. Wang C, Liu S, Zhang Y, Liu B, He F, Xu D, et al. Bacterial Communities and Their Predicted Functions Explain the Sediment Nitrogen Changes Along with Submerged Macrophyte Restoration.Microb.Ecol. 2018; 76: 625–636. https://doi.org/10.1007/s00248-018-1166-4

62. Zhang B, Zhang Y, Downing A, Niu Y. Distribution and Composition of Cyanobacteria and Microalgae Associated with Biological Soil Crusts in the Gurbantunggut Desert China. Arid Land Res.Manag. 2011; 25: 275–293. https://doi.org/10.1080/15324982011565858

63. Sheremet A, Jones GM, Jarett J, Bowers RM, Bedard I, Culham C, et al. Ecological and genomic analyses of candidate phylum WPS -2 bacteria in an unvegetated soil.Environ.Microbiol.2020; 22: 3143–3157. https://doi.org/10.1111/1462-292015054
